# Antibacterial Nanoplatelets via Crystallization-Driven Self-Assembly of Poly(l-lactide)-Based Block Copolymers

**DOI:** 10.1021/acs.biomac.4c00767

**Published:** 2024-08-06

**Authors:** Ahmad Alsawaf, Anne-Catherine Lehnen, Oleksandr Dolynchuk, Alain M. Bapolisi, Christina Beresowski, Alexander Böker, Ilko Bald, Matthias Hartlieb

**Affiliations:** † Institute of Chemistry, University of Potsdam, Karl-Liebknecht-Str. 24-25, 14476 Potsdam, Germany; ‡ Experimental Polymer Physics, Martin Luther University Halle-Wittenberg, Von-Danckelmann, Platz 3, 06120 Halle, Germany; § Fraunhofer Institute for Applied Polymer Research (IAP), Geiselbergstraße 69, 14476 Potsdam, Germany

## Abstract

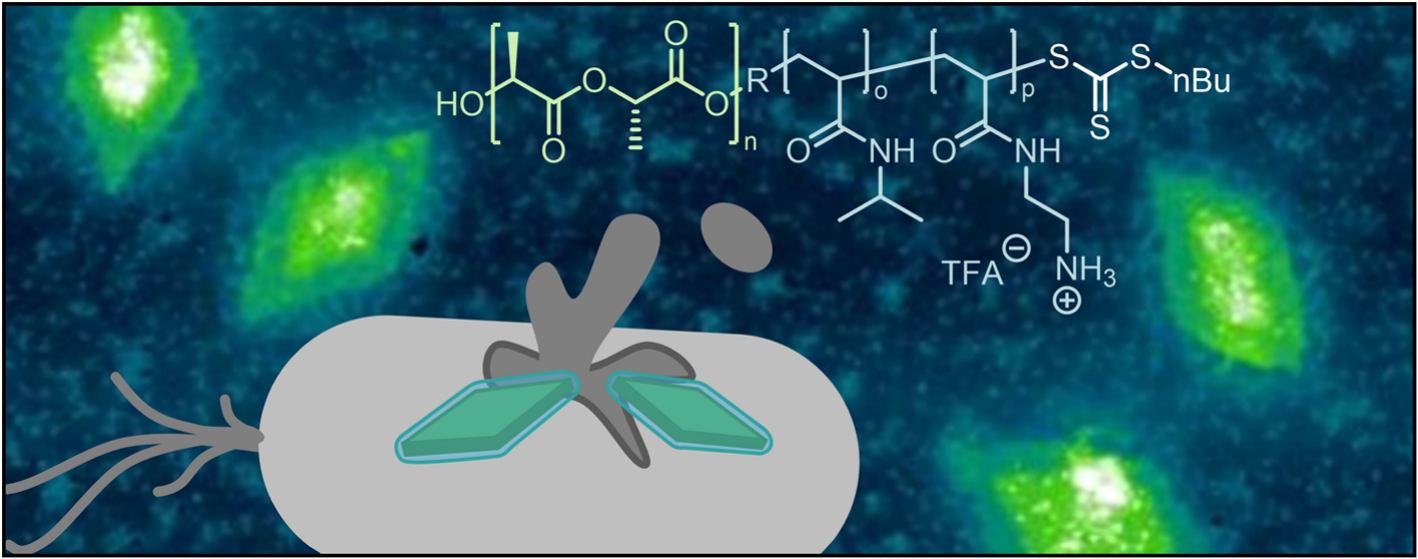

Membrane-active antimicrobial materials are promising substances to fight antimicrobial resistance. Herein, crystallization-driven self-assembly (CDSA) is employed for the preparation of nanoparticles with different morphologies, and their bioactivity is explored. Block copolymers (BCPs) featuring a crystallizable and antimicrobial block were synthesized using a combination of ring-opening and photoiniferter RAFT polymerizations. Subsequently formed nanostructures formed by CDSA could not be deprotected without degradation of the structures. CDSA of deprotected BCPs yielded 2D diamond-shaped nanoplatelets in MeOH, while spherical nanostructures were observed for assembly in water. Platelets exhibited improved antibacterial capabilities against two Gram-negative bacteria (*Escherichia coli* and *Pseudomonas aeruginosa*) compared to their spherical counterparts. The absence of hemolytic activity leads to the excellent selectivity of platelets. A mechanism based on membrane permeabilization was confirmed via dye-leakage assays. This study emphasized the impact of the shape of nanostructures on their interaction with bacterial cells and how a controlled assembly can improve bioactivity.

## Introduction

The development of antibiotics, such as penicillin,^1^ has paved the way for a plethora of medical progress in the past century. However, over- and misuse of conventional antibiotics has led to increasing levels of antimicrobial resistance (AMR).^2−4^ Predictably, 10 million people will die annually as a result of the emergence of drug-resistant bacteria by 2050.^5^ To find a solution to this dilemma, significant research efforts have been invested in the development of alternative antimicrobial compounds. In this context, silver nanoparticles,^6,7^ metal oxide nanoparticles,^8,9^ antimicrobial peptides,^10,11^ and antimicrobial polymers^12,13^ have stood out as potentially effective agents against microbes with variant mechanisms of action. However, inorganic-based nanoparticles can induce adverse side effects in mammalian cells and their mechanism of action is still under investigation.^14^ Antimicrobial peptides can be degraded by proteases, and their synthesis is usually costly.^15^ Still, nanoparticles decorated with cationic polymers have demonstrated their capability to suppress the growth and proliferation of bacteria owing to their high surface area, which leads to enhanced activity.^16^ As such, installing antimicrobial polymers on the surface of nanoscale materials seems like a worthwhile goal, and block copolymers (BCPs) that are able to assemble into nanostructures and feature antimicrobial polymer segments are promising building blocks. In addition to their antibacterial activity,^17^ they have a low chance of causing the development of new resistances.^18,19^

Intrinsically, cationic antimicrobial polymers contain two functional components namely cationic and hydrophobic subunits.^20,21^ These synthetic polycations possess a characteristically high charge density and can attach to the anionic bacterial cell membrane via electrostatic interactions with subsequent incorporation of the hydrophobic subunits into the lipid bilayer. However, when using nanostructured materials, the efficacy to disrupt the membrane is geometrically defined by the contact area between the nano-object and the membrane, as well as the local curvature of both at the contact point.^22^ Herein, various structural merits of polycationic nanostructures have been methodically adjusted to enhance their antimicrobial activities by altering their chemical nature, dimension, shape, and surface charge.^23−25^ A versatile way to control the shape of nano-objects based on BCP is the use of semicrystalline polymers in crystallization-driven self-assembly (CDSA).^26−29^ While crystallites grow in 3D, the attachment of a second block can restrict the growth to 1D nanofibers or 2D nanoplatelets, yielding nanofibers or platelets, respectively. Moreover, controlling the dimension of such objects has also been accomplished through seeded growth methods (also called living CDSA) and has been widely applied for BCPs to form cylindrical structures,^30−36^ as well as platelets.^37−40^

In the context of antimicrobial materials, Jang and colleagues illustrated an enhanced antibacterial activity upon decreasing the diameter of spherical nanoparticles as a result of increasing their surface area.^41^ However, 1D nanoparticles have been renowned for having benefits relative to other morphologies in a living organism in terms of prolonged persistence in the body because of increased circulation times and reduced renal clearance and macrophage uptake.^42−44^

O’Reilly and co-workers used nanostructures derived from BCPs of poly(l-lactic acid) (PLLA) and poly(dimethyl aminoethyl acrylate) (PDMAEMA).^25^ They investigated the impact of size and shape on the antimicrobial activity of the materials obtained by CDSA after quaternization. Small platelets performed better than large structures and spherical assemblies. Moreover, O’Reilly and team explored the performance of monodisperse cationic cylindrical micelles prepared by living CDSA, where the polycationic cylinders presented slightly improved antibacterial properties against Gram-negative and -positive bacteria that did not follow a clear correlation with fiber length.^16^ Recently, Manners and co-workers reported the synthesis of uniform antibacterial nanofibers based on the BCP of poly(fluorene trimethylene carbonate) (PFTMC)_16_-*b*-PDMAEMA_131_ via living CDSA.^45^ It was found that the performance of the nanofibers was length-dependent, where longer nanofibers had higher antibacterial activity against *Escherichia coli* compared to intermediate-length and short nanofibers. In addition, the nanofibers outperformed their nanosphere counterparts at each investigated length. In more detailed investigations, it was shown that initial attachment to the membrane is not strongly affected by the shape of nano-objects but that intercalation into the membrane is more pronounced for rigid 1D fibers.^15^

The results from the groups of O’Reilly and Manners emphasize an impact of shape on the antimicrobial activity. However, the reported systems were based on tertiary or quaternary amino groups, in part combined with relatively hydrophobic substituents. Such materials usually face difficulties with their compatibility regarding mammalian cells,^20^ a quality that was not tested in these studies. However, knowledge about biocompatibility is essential to determine the selectivity for bacterial cells over host cells.

In our previous work, we have demonstrated how crucial the shape and anisotropy of antimicrobial polymers is on a molecular level.^46,47^ The limited and modulated hydrophobicity of these systems based on *N*-isopropylacrylamide (NIPAM) and amino ethyl acrylamide (AEAM) leads to impressive selectivities. Thus, we aimed to probe if these polymers can be applied as a component in antimicrobial nanostructures via CDSA. The anisotropic nature of such assemblies could enhance the activity of the system as indicated by the studies by O’Reilly and co-workers discussed above. We were able to show that Boc-protected precursors can be produced using a combination of ring-opening polymerization (ROP) and photoiniferter reversible addition–fragmentation chain-transfer (PI-RAFT) polymerization.^48^ Herein, we discuss strategies to produce antimicrobial materials from these precursors and test their performance against common pathogenic bacteria strains.

## Results and Discussion

### Synthesis of Diblock Copolymers

**Scheme 1:**
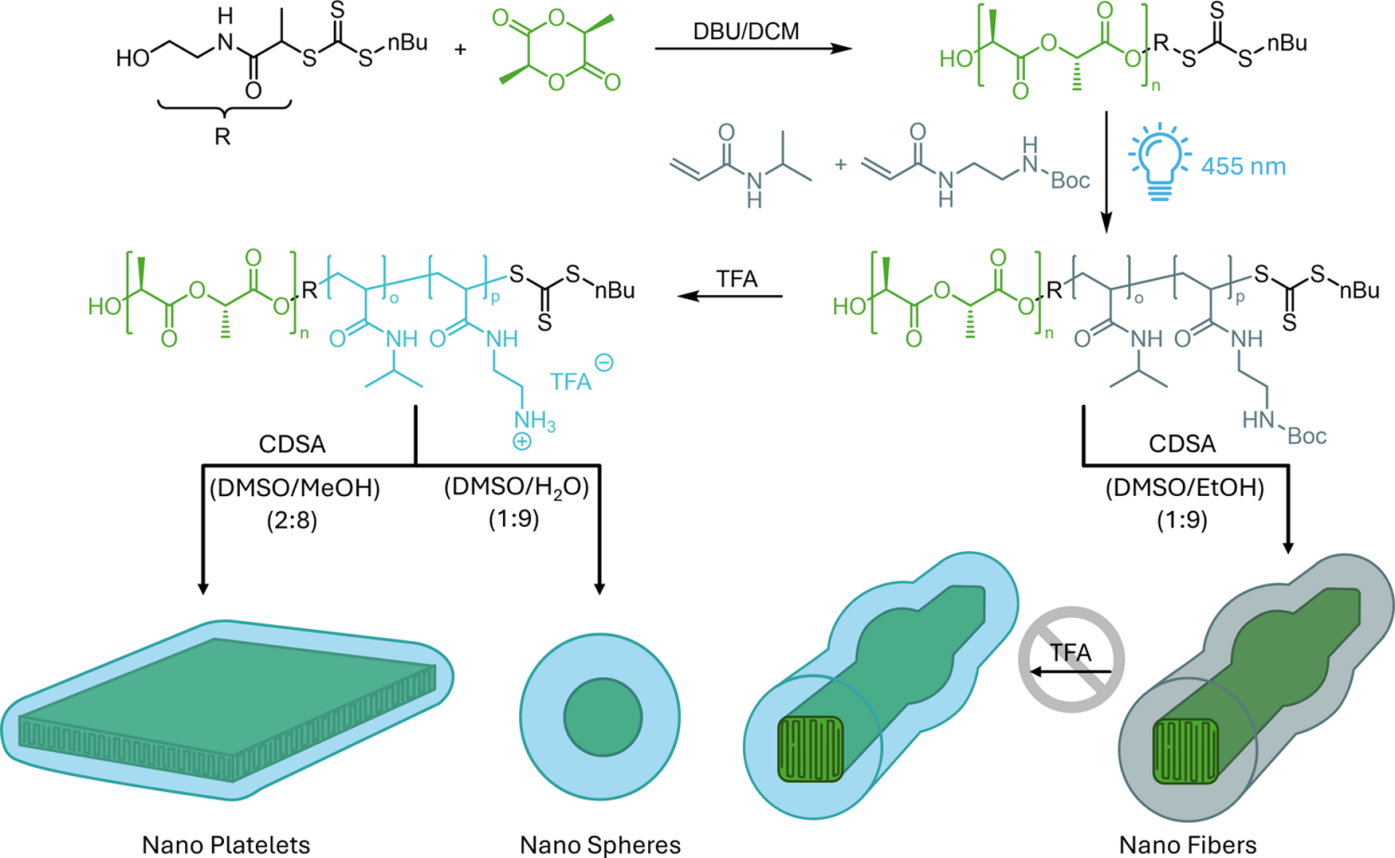
Synthesis of BCP by ROP of l-Lactide Using a Hydroxy-Functional CTA and Subsequent Chain Extension of This Macro-CTA with BocAEAM and NIPAM Schematic representation of CDSA of protected and deprotected BCPs to form various nanostructures.

According to the synthetic strategy established previously,^48^ amphiphilic BCPs possessing the general composition of PLLA*_n_*-*b*-P(BocAEAM_o_-*co*-NIPAM_p_) were successfully synthesized using a combination of ROP and PI-RAFT polymerization. First, a PLLA macro-chain transfer agent (CTA) was synthesized by ROP of l-lactide using a hydroxyl-functionalized CTA as an initiator and diazabicycloundecen (DBU) as a catalyst. Subsequently, the prepared macro-CTA was activated via blue light (λ = 455 nm, *W* ∼ 200 mW cm^–2^) in the presence of the monomers BocAEAm and NIPAM (Scheme 1). A PI-RAFT strategy^49^ was employed as it was demonstrated previously that chain extension proceeds in a more controlled way compared to conventional RAFT polymerization.^48^

While PLLA was chosen due to its semicrystalline properties to form the core of later nanostructures, BocAEAM and NIPAM were selected because of their antimicrobial properties after deprotection. The comonomer selection was based on previous investigations of their antimicrobial performance.^50,51^ NIPAM was used as it exhibits moderate hydrophobicity, leading to materials with a low hemolysis and low cytotoxicity against human cells. Two different corona lengths of P(BocAEAM_o_-*co*-NIPAM_p_) with an approximate DP of 300 and 600 were targeted by PI-RAFT polymerization using a macro-CTA of PLLA_50_-CTA. This was to probe the influence of BCP composition on later CDSA. ^1^H NMR spectroscopic analysis was performed to determine the respective DPs of the crystallizable PLLA block (Figure S1) and the respective BCPs (Figures S2 and S3). A DP of 50 was chosen for PLLA to ensure sufficient block length of the semicrystalline part to create anisotropic structures during CDSA.

**Figure 1:**
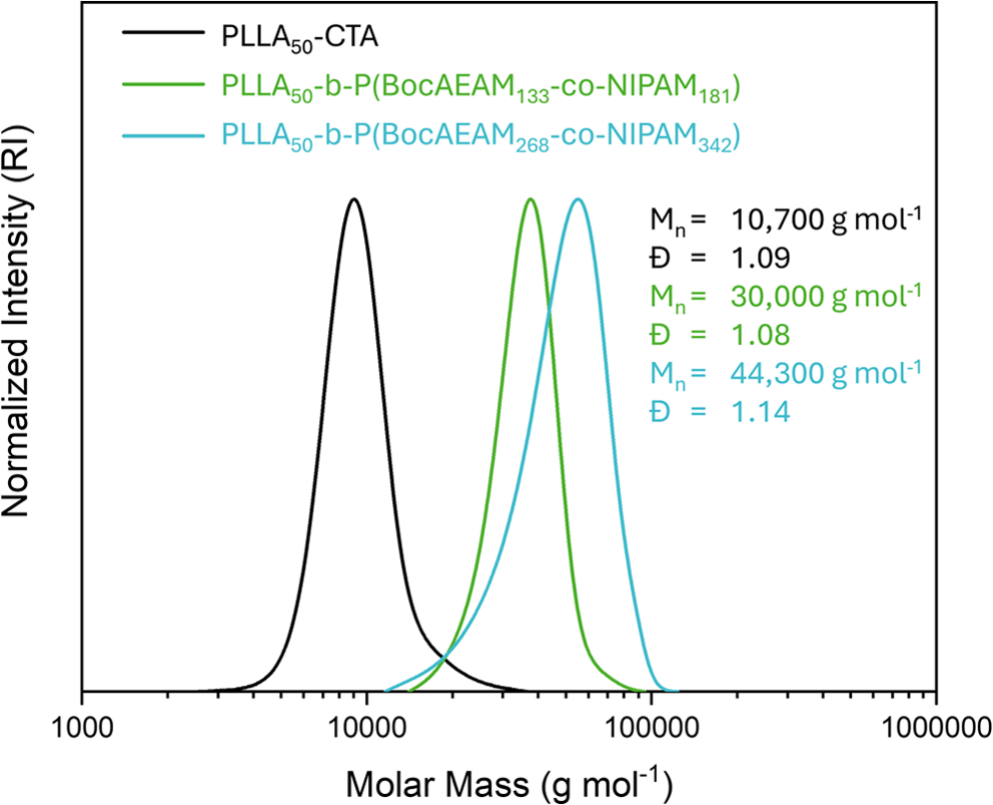
Overlay of SEC traces of PLLA_50_-CTA as well as PLLA*_n_*-*b*-P(BocAEAM*_o_*-*co*-NIPAM*_p_*) with two different corona lengths (DP of 314 and 610) obtained via PI-RAFT polymerization and measured by SEC in THF using a poly(styrene) (PS) calibration.

Experimental DPs for PLLA (DP = 50) and corona-forming blocks (DP = 314 and 610) were in line with the targeted theoretical values. Moreover, unimodal distributions with a controlled dispersity of 1.1 for the PLLA core and its corona-forming block were observed via SEC analysis (Figure 1). The SEC traces of BCPs indicated noticeable shifts relative to the SEC trace of the crystallizable core of PLLA_50_.

The characterization data of the crystallizable core and the respective BCPs are also displayed in Table 1. An approximate ratio of 40% BocAEAM and 60% NIPAM for both BCPs was determined, which will later result in an amphiphilic balance suitable for targeting bacterial cell membranes.^50^

**Table 1:** Characterization Data of Macro-CTA and BPSs

sample	conv.^a^ (%)	DP_total_^a^ (block)	*M̅*_n_^b^ (g mol^–1^)	*Đ*^b^ (−)	BocAEAM^a^ (%)	NIPAM^a^ (%)
macro-CTA of PLLA_50_-PABTC–OH	≥99	50	10 600	1.10		
PLLA_50_-*b*-P(BocAEAM_133_-*co*-PNIPAM_181_)	98	314	34 500	1.06	42	58
PLLA_50_-*b*-P(BocAEAM_268_-*co*-NIPAM_342_)	98	610	44 300	1.14	44	56

a Determined via ^1^H NMR spectroscopy in DMSO-*d*_6_.

b Determined via SEC of RI detection in THF using a PS calibration.

### CDSA of PLLA*_n_*-*b*-P(BocAEAm*_o_*-*co*-NIPAM*_p_*) Diblock Copolymers

**Figure 2:**
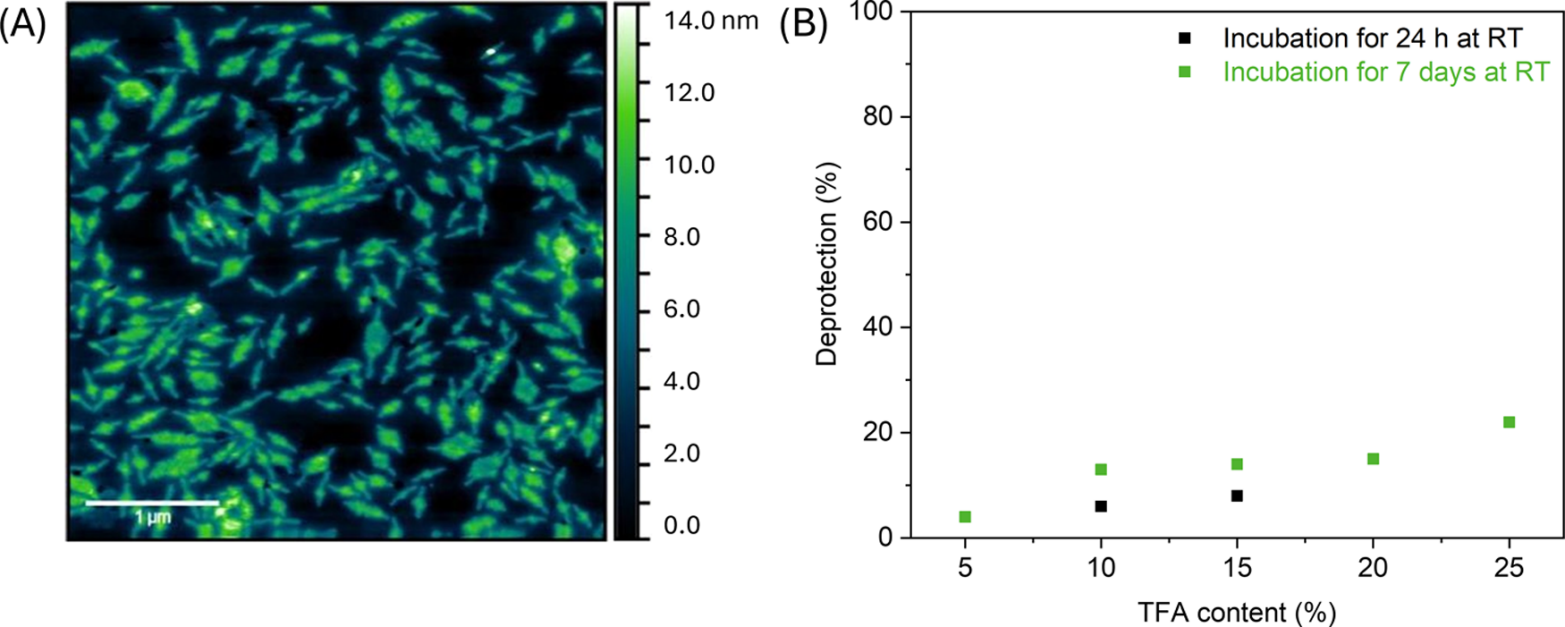
(A) AFM height image of diblock copolymers of PLLA_50_-*b*-P(BocAEAM_133_-*co*-NIPAM_181_) of polydisperse nanostructures deposited from a diluted dispersion of a concentration of 0.5 mg mL^–1^. (B) Boc deprotection of the nanostructures of PLLA_50_-*b*-P(BocAEAM_133_-*co*-NIPAM_181_) determined via ^1^H NMR spectroscopy.

The formation of polydisperse nanostructures was achieved via the direct dissolution method adapted from O’Reilly’s group.^48,52^ Assemblies were prepared via spontaneous nucleation using a mixture of DMSO/EtOH (v/v = 1:9) at a polymer concentration of 5 mg mL^–1^, as shown in Scheme 1. Atomic force microscopy (AFM) analysis was used to assess the structure and dimensions of the formed nanostructures (Figures 2A and S4). As observed previously, bulging in the center of each structure indicates a tendency to grow into more than one dimension. BCPs are likely in between fiber forming and platelet forming in regard to the block ratio.

To generate antimicrobial nanostructures, the produced objects were subjected to a deprotection process to remove the Boc-protection group of the BocAEAm units. The Boc-deprotection process was performed by incubating the dispersion with different concentrations of TFA (between 5 and 25%) for 24 h or 7 days at room temperature (Figure 2B). ^1^H NMR spectroscopy was used to determine the percentage of deprotection, and dynamic light scattering (DLS) analysis was implemented to measure the average size of the treated nano-objects. A summary of ^1^H NMR data and DLS analysis of the Boc deprotection process from the polydisperse nano-objects with different conditions is shown in Table S1.

Overall, a low percentage of Boc-deprotection concomitant with PLLA core degradation was observed by ^1^H NMR spectroscopy. A solvent change to water led to the visible precipitation of nanostructures. Even treatment with a relatively high concentration of 75% TFA with a short incubation time of 1 h was not sufficient to increase the percentage of Boc-deprotection to more than 20%, as proved by ^1^H NMR spectra (Figure S6). These results show that deprotection after CDSA is not a viable strategy for antimicrobial nanomaterials based on the presented materials.

**Figure 3:**
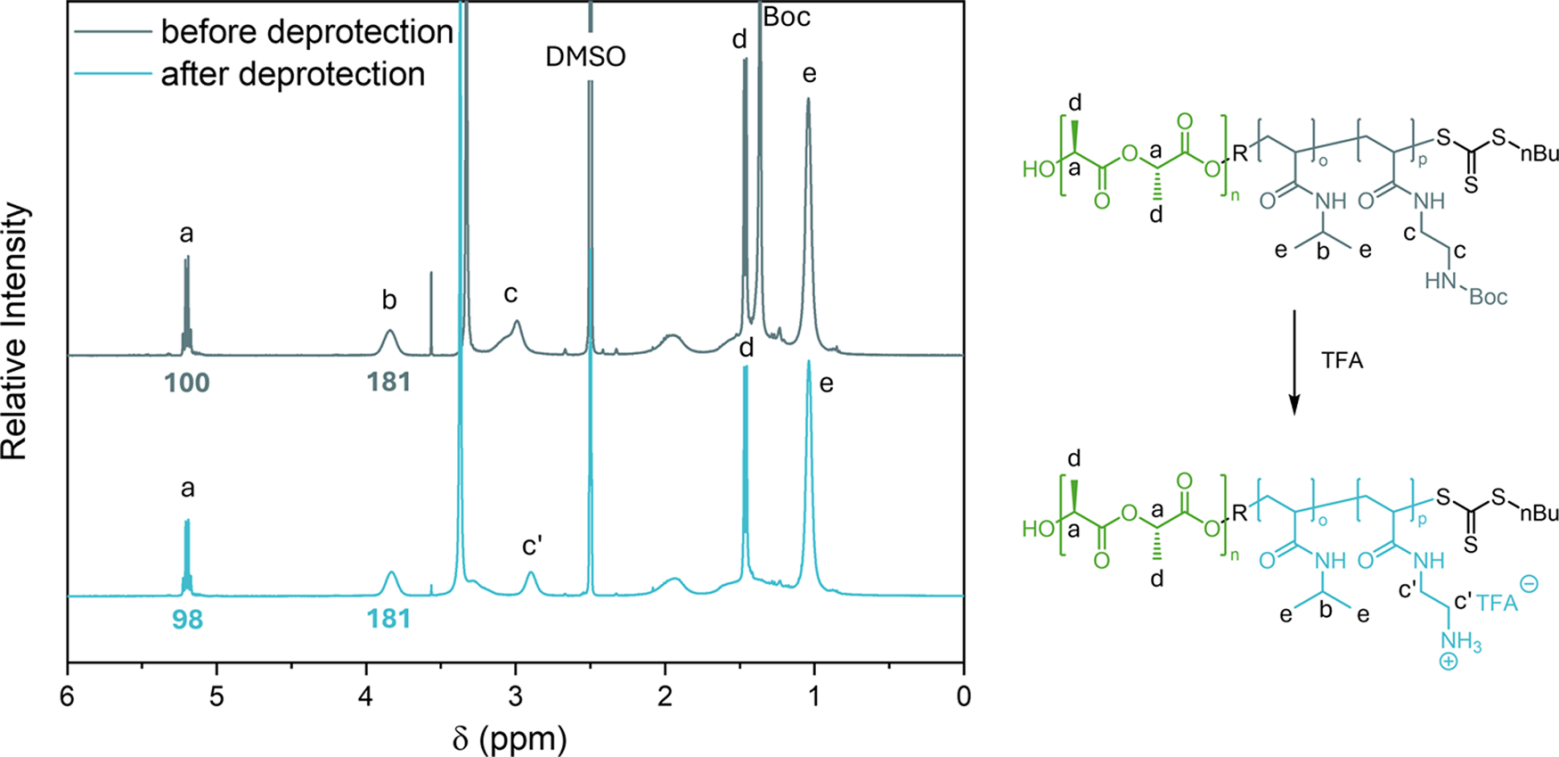
Boc deprotection process of PLLA_50_-*b*-P(BocAEAM_133_-*co*-PNIPAM_181_) via treatment with TFA at RT for 20 min was analyzed by ^1^H NMR spectroscopy in DMSO-*d*_6_.

As an alternative pathway, the original precursor PLLA_50_-*b*-P(BocAEAM_133_-*co*-NIPAM_181_) was subjected to deprotection prior to crystallization. The corresponding BCPs were directly treated with TFA in a concentrated solution of 100 mg mL^–1^ at room temperature for 20 min (Figure 3). As a result, full Boc-deprotection was achieved as determined by ^1^H NMR spectroscopy. In addition, the –C***H***(CH_3_)_2_ of NIPAM and the O–C***H***(CH_3_)–CO signal of PLLA were compared before and after deprotection, revealing no significant levels of degradation.

**Figure 4:**
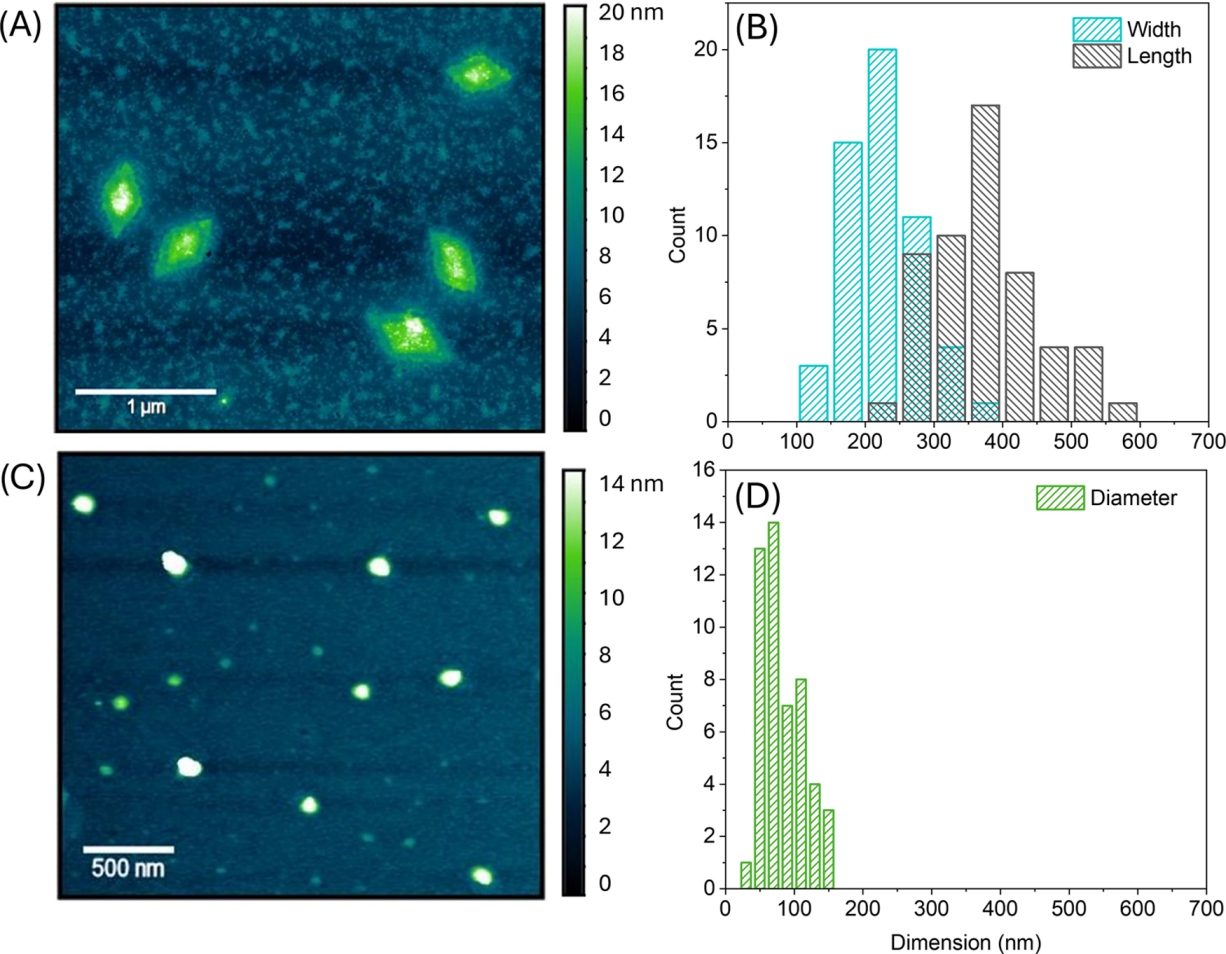
AFM height image of nano-objects formed by CDSA of PLLA_50_-*b*-P(AEAM_133_-*co*-NIPAAM_181_) in DMSO/MeOH (2:8) (A), or DMSO/water (1:9) (C) deposited from a diluted dispersion after dialysis against PBS at a pH of 7.5 at a concentration of 0.5 mg mL^–1^. Multiple images (54 for platelets, 50 for spheres) were used to measure the size of platelets (B) and spheres (D).

To create anisotropic nanostructures (fibers or platelets) that show improved antimicrobial activity compared to that of isotropic nanostructures (spheres), different methods were applied for self-assembly. To induce CDSA, polymers were dissolved in DMSO, which was subsequently mixed with MeOH to enable crystallization over a period of days. In contrast, when using water as a cosolvent, the faster self-assembly process did not allow for crystallization. Thus, after successful deprotection of polymers, CDSA was applied by annealing 5 mg mL^–1^ BCPs (PLLA_50_-*b*-P(BocAEAM_133_-*co*-PNIPAM_181_)) in DMSO/MeOH (v/v = 2:8) at 60 °C for 3 h before cooling to room temperature. Surprisingly, AFM analysis revealed 2D diamond-shaped platelets (as opposed to ill-defined objects before deprotection) after 1 week of aging the solution (Figures 4A and S7). Upon changing the solvent mixture to DMSO/H_2_O (v/v = 1:9), spherical nanostructures were also observed by annealing 5 mg mL^–1^ deprotected BCPs at 85 °C for 4 h after 3 days of aging by AFM (Figures 4C and S7).

As can be seen from the AFM cross-section, a thickness of 6 nm was detected for three separated nanoplatelets (Figure S13), whereas a thickness of about 13 nm was revealed for their spherical counterparts (Figure S14). The nonuniform height profile of the cross-section of the platelets can be explained by the high DP of the corona-forming block, which is dried around the actual semicrystalline core. The length of the formed platelets was measured along the longest axis with an average length of 358 ± 102 nm, while the width was determined with 219 ± 99 nm, measuring the shorter axis of the platelets (Figure 4B). A diameter of about 74 ± 40 nm was determined for the spherical objects (Figure 4D).

DLS measurements were conducted to determine the hydrodynamic diameters of the generated nanostructures (Figure S10). A monomodal size distribution of the corresponding nanoplatelets with a *z*-average of 319 nm and a polydispersity index (PDI) of 0.22 was observed after dialysis against PBS. However, a *z*-average of 381 nm and a PDI of 0.57 as well as a bimodal distribution were found for spherical objects, indicating aggregation. Furthermore, positive ζ-potential values were obtained for both types of nanostructures (+14.0 for platelets and +17.3 for spheres), indicating the presence of cationic units on the interfaces.

To investigate the influence of the length of the corona-forming block on the morphology, CDSA in DMSO/MeOH (v/v = 2:8) was performed for the longer BCPs with a DP of 610. However, the self-assembly behavior of the deprotected PLLA_50_-*b*-P(AEAM_268_-*co*-PNIPAM_342_) did not result in the formation of uniform morphologies (AFM in Figure S11 and DLS in Figure S12) and partial precipitation was observed after aging.

**Figure 5:**
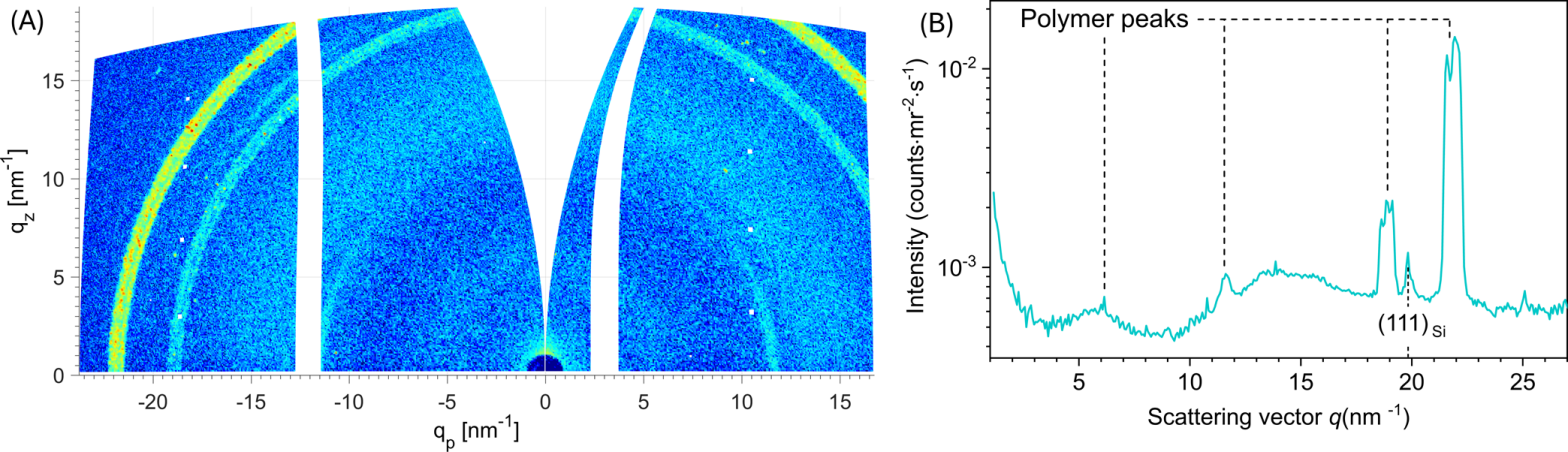
GIWAXS pattern (A) and corresponding scattering curve (B) of the drop-cast sample on the silicon substrate measured at an incident angle of 0.2°. The scattering curve in (B) was obtained by integrating the intensity in (A) over all azimuthal angles.

To determine if samples were crystalline, grazing-incidence wide-angle X-ray scattering (GIWAXS) was measured on the drop-cast sample of nanoplatelets on a silicon wafer. The angle of incidence was 0.2°, which is below the critical angle of the silicon substrate. Such a choice of angle of incidence allows the scattering from the substrate to be minimized and the scattering from the polymer to be enhanced. As seen in Figure 5, the scattering signal from the polymer is clearly visible and can be well separated from the weak (111) peak from the silicon substrate. We identified three well-defined polymer peaks at *q* = 11.6, 18.9, and 21.8 nm^–1^ and a broad diffuse scattering signal at about *q* = 5.7 nm^–1^. The additional measurement performed at the same angle of incidence but near the substrate edge (Figure S15), where almost no polymer could be detected optically shows only a very faint peak at a *q* of 21.8 nm^–1^. Thus, we readily conclude that the peaks observed in Figure 5 are indeed scattered by the polymer crystallites. The additional polarized optical and atomic force microscopy measurements (Figure S16) show that the polymer is semicrystalline, confirming the GIWAXS results.

### Bioactivity of Nanostructures

**Figure 6:**
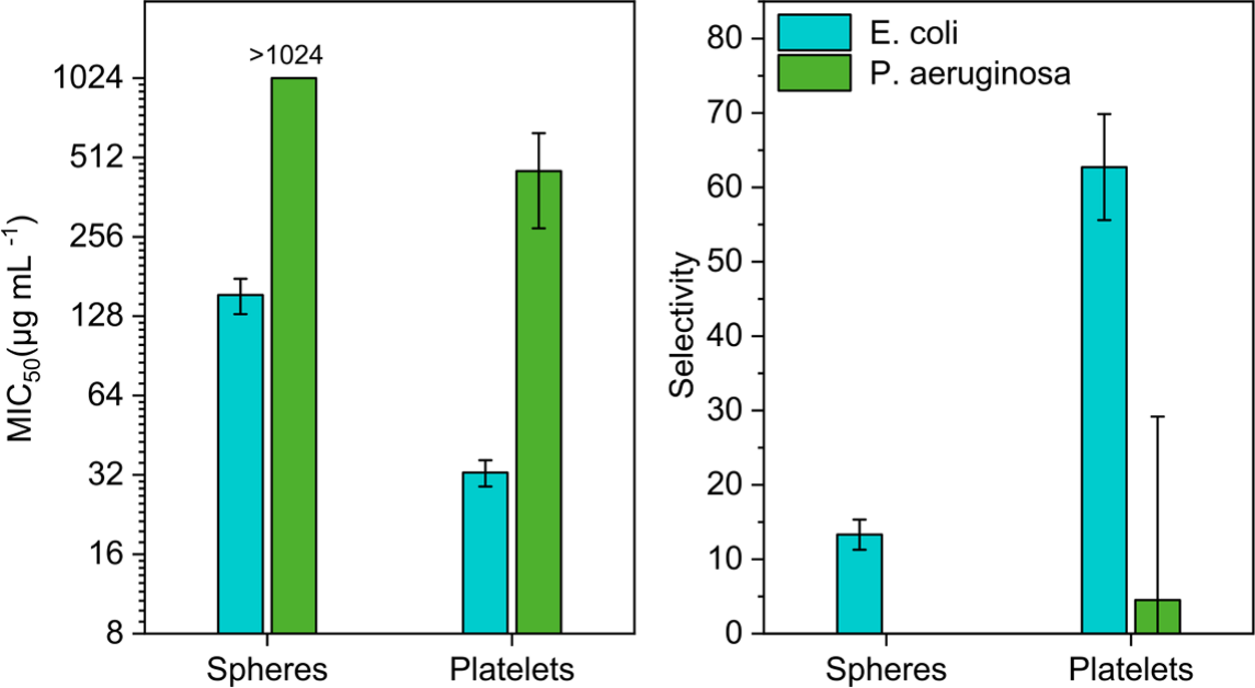
MIC_50_ values of cationic nano-objects as determined by growth inhibition studies against *E. coli* and *P. aeruginosa* (data for MRSA not shown as no growth inhibition was observed) based on the Hill1 fit of concentration-dependent data using OriginPro 2021. Selectivity was determined using a hemolytic concentration (HC_10_).

To probe the antibacterial activity of the nanostructures, three relevant pathogenic strains of bacteria including two Gram-negative bacteria, *Escherichia coli*, *Pseudomonas aeruginosa*, and one Gram-positive strain, methicillin-resistant *Staphylococcus aureus* (MRSA), were tested. Antimicrobial activity was assessed by measurement of the minimal inhibitory concentration (MIC_50_) against bacteria in suspension (Figure S17). Hemocompatibility was probed against red blood cells (RBCs) by determining the hemolytic concentrations (HC_10_). Based on these values, a selectivity (= HC_10_/MIC_50_) can be derived to quantify the performance of the materials. The results of the tests are summarized in Figure 6 and Table 2.

**Table 2:** Summary of Characterization Data for BCP-Derived Nanostructures

		a			MIC_50_^b^ (μg mL^–1^)				selectivity		
samples	*Z*-average^a^ (nm)	PDI^a^	ζ^a^ (mV)	Δζ^a^ (mV)	EC^b^	PA^b^	SA^b^	HC_10_ (μg mL^–1^)	EC^b^	PA^b^	SA^b^
platelets	319	0.22	+14.0	1.13	33 ± 4	454 ± 178	>1024	>2048	63 ± 7	5 ± 25	
spheres	381	0.57	+17.3	0.47	154 ± 24	>1024	>1024	>2048	13 ± 2		

a Measured via DLS after dialysis against PBS at pH of 7.5 at a concentration of 1 mg mL^–1^ in ultrapure water.

b Bacteria abbreviations: EC = *E. coli*, PA = *P. aeruginosa*, and SA = *S. aureus*.

While both types of nanostructures are inactive against Gram-positive MRSA, they are active against *E. coli* and to some extent against *P. aeruginosa*. The inability of nano-objects to permeabilize the membrane of MRSA could be associated with the thick peptidoglycan layer of Gram-positive bacterial strains, which could prevent nano-objects in the size range of the materials presented herein from reaching the cellular membrane. Likewise, *P. aeruginosa* is known to readily secrete the biopolymer into the extracellular space that could potentially trap nanoscopic objects.^53^ However, the shape and size of cationic nano-objects have a significant influence on their activity. While spheres only show moderate activity against *E. coli* and no activity against *P. aeruginosa*, nanoplatelets show good antimicrobial activity against *E. coli* and measurable growth inhibition against *P. aeruginosa*. It should be noted that both structures are based on the same BCP and have identical compositions of the polymer shell. Also, the zeta potential and size are in a similar range. What seems to make the difference is the shape of the structure with defined platelets revealing a larger surface area when compared with spherical structures. While the size varies between spheres and platelets, they are both in the same size range, being significantly larger than conventional antimicrobial polymers or peptides while still being smaller than bacteria. From the perspective of the bacterial cell envelope, the different curvatures between spherical and flat nano-objects are likely the most influential parameter. The similarity of the interaction kinetics with liposomes (vide infra) indicates this as well.

Hemolysis was tested using defibrinated sheep blood, and red blood cells (RBCs) were isolated to examine the toxicity of cationic nanostructures (Figure S18). No hemolytic activity was detected for both structures within the investigated concentration range (up to 2048 μg mL^–1^). This finding is in line with our previous studies on copolymers that are herein used as a polymer shell.^50^ Accordingly, the selectivity of the nanoparticles was calculated by dividing the HC_10_ value by the MIC_50_ for the respective bacterium strain. Also, here, the positive impact of the flat platelet morphology was obvious as an excellent selectivity value of 63 was achieved against *E. coli*.

In comparison with molecular bottle brush copolymers featuring a relatively rigid backbone (poly(norbornene)-based) and a comparable composition,^50^ the MIC against *E. coli* is slightly improved for platelets (33 vs 64 μg mL^–1^), while neither of the materials are hemotoxic. However, it should be noted that in the present study, grafts are substantially longer. As we have also shown that the degree of freedom of side chains has a severe impact on bioactivity,^46^ a direct comparison might not be overly meaningful. Another difference lies in the selectivity between the bacteria strains. While molecular bottle brushes are active against Gram-positive and -negative bacteria alike,^47^ the larger size of nano-objects produced by CDSA seems to favor Gram-negative strains. Such selective activity based on the size, in combination with the improved activity based on the anisotropic shape, could be interesting for targeted eradication of pathogenic bacteria.

### Dye Leakage Assays Using Liposomes

**Figure 7:**
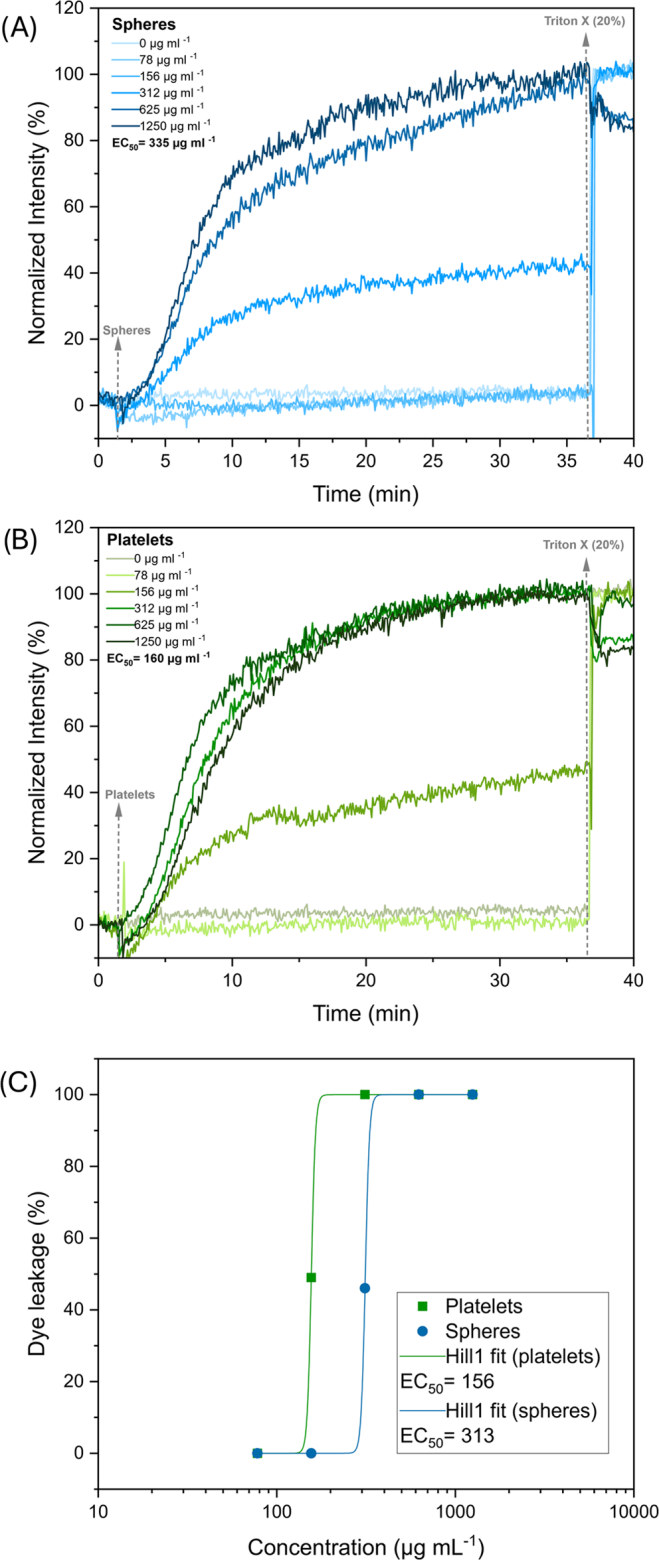
Dye leakage from *E. coli* mimicking liposomes (0.125 mg mL^–1^) induced by spheres (A) and platelets (B). The initial baseline corresponds to 0% of normalized dye leakage intensity before adding the respective nanostructures. The change in the fluorescence caused by the nano-objects in a concentration-dependent manner was monitored over time up to equilibrium. Triton *X* was added at the end as a positive control to disrupt all of the liposomes and to reflect the maximum (100%) dye leakage. Data were fitted with a Hill1 function (C) in OriginPro 2021.

To confirm that the antimicrobial activity of the nanostructures is based on a membrane permeabilization mechanism, dye leakage assays using liposomes that mimic the membrane composition of *E. coli* were performed. Noticeably, both spheres (Figure 7A) and platelets (Figure 7B) induce dye leakage in a concentration-dependent manner, with up to 100% of dye liberated at high concentrations after 30 min of incubation. The main difference was found in the EC_50_ values (effective concentration to induce 50% of dye leakage, Figure 7C). Here, platelets (EC_50_ = 156 μg mL^–1^) were more effective than the spheres (EC_50_ = 303 μg mL^–1^), reflecting a similar tendency found for MIC_50_ values against *E. coli*. This demonstrates that direct membrane permeabilization is guided by the shape of the cationic nano-object used in antibacterial applications. The time frame necessary for equilibration is also an interesting outcome of this test. While it does not markedly vary between differently shaped nanostructures, leakage is significantly delayed when compared to bottle brush copolymers with a similar composition of the active polymer shell.^47^ In comparison, the total time of the assay had to be increased from 10 to 40 min to capture the entire process. One possible explanation would be the decreased diffusivity of nano-objects when compared to bottle brush copolymers due to differences in size.

## Conclusions

In summary, the preparation of cationic nanoplatelets via CDSA and their application as an antimicrobial material are shown. Polymers are based on PLLA constituting the crystallizable block and possess a shell composed of a mixture of NIPAM and BocAEAM featuring a Boc-protected primary amine group. We explored two routes toward cationic nanostructures using these BCPs. The assembly of Boc-protected copolymers led to structures with a fibrous morphology. However, it was demonstrated that removing the protection group also degrades the assembled structures. In an alternative process, BCPs were deprotected, and CDSA was performed with cationic copolymers. This led to the formation of well-defined nanoplatelets or spherical structures based on the conditions during self-assembly. Both morphologies were similar in size and ζ-potential and were found to be semicrystalline, as revealed by GISAXS measurements.

The bioactivity of the nano-objects was probed via growth inhibition assays against different bacterial strains. While no growth inhibition was detected against MRSA with only limited activity against *P. aeruginosa*, platelets were highly active against *E. coli*. Moreover, the platelet morphology showed significantly improved activity and selectivity relative to spherical nanostructures featuring the identical composition. A mechanism based on membrane permeabilization was confirmed by dye leakage studies using model membranes mimicking *E. coli*. Also, in this model, platelets clearly outperformed the spherical structures. Here, it was also found that permeabilization by nano-objects is much slower than for smaller polymeric structures with a similar composition.

These findings highlight the importance of shape in membrane-active antimicrobial nanomaterials and demonstrate that highly selective nanomaterials can be constructed via the CDSA of BCPs.

## Experimental Section

### Materials and Methods

All chemicals and solvents were used as received without further purifications unless otherwise stated and purchased from Sigma-Aldrich, Alfa-Aesar, Carl Roth Co., Merck, TCI (Tokyo chemical industry), Acros Organics, and Fisher Chemicals: 3s,6s-3,6-Dimethyl-1,4-dioxane-2,5-dione (l-(−)-lactide), 1,8- diazabicyclo[5.4.0]undec-7-ene (DBU), dry dichloromethane (DCM), trifluoroacetic acid (TFA), methanol (MeOH), ethanol (EtOH), dimethyl sulfoxide (DMSO), 1,4-dioxane, diethyl ether (Et_2_O), phosphate-buffered saline (PBS), Mueller–Hinton broth medium (MHB), Triton X solution, 2-oleoyl-1-palmitoyl-*sn*-glycero-3-phosphoethanolamine (POPE), 2-oleoyl-1-palmitoyl-*sn*-glycero-3-phospho-rac-(1-glycerol) sodium salt (POPG), chloroform (CHCl_3_), and calcein solution. The inhibitor from *N*-isopropylacrylamid (NIPAM) was removed by passing through a glass Pasteur pipet of neutral Al_2_O_3_.

A PhotoCube from ThalesNano was used to conduct photopolymerizations. The photoreactor was cooled through cooling water to maintain a temperature of 20 °C. For polymerizations, only 455 nm light was applied at a high setting intensity of 100%. The LED intensity was measured at the sample position with a commercial S170C power meter (Thorlabs). It should be noted that the flat sensor fits into the sample chamber only vertically, while the reaction chamber is illuminated from all four sides. Hence, 1/4 of the LEDs are behind the sensor. This was adjusted by setting the measured intensity to be 3/4 of the total intensity with an approximation of 204 mW cm^–2^.

NMR spectroscopic measurements were recorded on a Bruker Avance III-HD 400 spectrometer operating at 400 MHz for ^1^H NMR spectroscopy at room temperature. All chemical shifts (δ) are reported in parts per million regarding solvent residual signals of the deuterated solvent (CDCl_3_, DMSO-*d*_6_) as the internal reference. The following reference values of the deuterated solvents were used: CDCl_3_ (^1^H NMR: δ = 7.26 ppm, singlet) and DMSO-*d*_6_ (^1^H NMR: δ = 2.50 ppm, quintet). The spectral data were analyzed by using TopSpin software.

Size exclusion chromatography (SEC) measurements were conducted with simultaneous UV and RI detection using THF as an eluent with a flow rate of 0.5 mL min^–1^ at room temperature; the stationary phase was a 300 × 8 mm^2^ PSS SDV linear M column.

Atomic force microscopy (AFM) samples were prepared by drop-casting 5–7 μL of diluted assemblies of 0.5 mg mL^–1^ using a micropipette onto a silicon wafer (for neutral nanostructures) and freshly cleaved mica (for cationic nanoplatelets and nanospheres) followed by quick drying by applying a vacuum. Imaging and analysis were performed using the state-of-the-art OmegaScope scanning probe microscope (Horiba Scientific, France) through head HE002. The samples were measured by applying tapping mode using commercial tips purchased from Budget Sensors (Tap-150Al-G, 10 nm radius) with a resonance frequency of 150 kHz and a force constant of 5 N m^–1^. Opensource Gwyddion 2.59 software was used for the AFM image processing. ImageJ software was used for measuring the length and width of nanoplatelets (*n* = 54) and the diameter of nanospheres (*n* = 50). The count distribution histogram was fitted with a multiple peak fit using the Gauss function in OriginPro 2021.

DLS was used to measure the average size (*Z*-average) and polydispersity index (PDI) of the nanoparticles at 25 °C using a Malvern Zetasizer Ultra instrument equipped with a 4 mW laser module (633 nm) at a detection angle of 173°. DLS samples were analyzed in disposable plastic cuvettes of 10.0 mm path length with a concentration of 1 mg mL^–1^ and measured repeatedly three times.

ζ-Potentials were also obtained by using a Malvern Zetasizer Ultra by measuring the electrophoretic movement of the nanoparticles under an applied electric field at 25 °C. The samples were measured in a special capillary cuvette at a concentration of 1 mg mL^–1^ in PBS at a pH of 7.5.

A Fluoromax 4 spectrofluorometer (Horiba, USA) was used to monitor the change in fluorescence intensity of calcein. The excitation wavelength was set at 490 nm (slit: 1.0 nm bandpass), and the fluorescence intensity was monitored over time (2400 s) in which the CPS signals were recorded every 5 s at the emission wavelength of 525 nm (slit: 1.0 nm bandpass).

### Synthesis of *n*-Butyl 2-(2-hydroxyethylamino)-1-methyl-2-oxoethyl trithiocarbonate (PABTC–OH)

A hydroxyl functional CTA of PABTC–OH was synthesized following a similar method reported in our previous paper.^48^

### Synthesis of a Macro-CTA of PLLA_50_-PABTC–OH

The ring-opening polymerization of l-lactide initiated by PABTC–OH with a DP of 50 was performed according to a similar published procedure in the literature.^54^ First, l-lactide was dried gently using a preheated oil bath at 45 °C for 3 h under vacuum. A Schlenk tube equipped with a magnetic stirrer was used to transfer the dried l-lactide (321 mg, 2.23 mmol, 50 equiv). Then, PABTC–OH (13 mg, 0.045 mmol, 1 equiv) was added under an argon stream. After that, the starting materials were dissolved in a dry DCM (4 mL) at a concentration of 0.5 mol L^–1^. Subsequently, 0.2 equiv of DBU as a catalyst (1.5 μL, 0.01 mmol) prepared from a 10 wt % stock solution was added, and the resultant mixture was stirred for 3 h at room temperature. The reaction solution was quenched in cold methanol, and the precipitated polymer was isolated and collected by centrifugation. The polymer was obtained as a yellowish powder and dried overnight under a vacuum. ^1^H NMR (400 MHz, CDCl_3_) δ (ppm): δ = 6.64 (t, 1H, O-(CH_2_)_2_–N***H***-CO−); 5.19–5.13 (q, 51.3H, O–C***H***(CH_3_)-CO); 4.74–4.68 (q, 1H, C***H***(CH_3_)–CONH); 4.21–4.18 (t, 2H, O–C***H***_***2***_–CH_2_–NH–CO−); 3.61–3.32 (m, 4H, C***H***_***2***_–NH–CO–, C***H***_***2***_-S-CS_2_); 1.58–1.57 ppm (m, 157H, O–CH(C***H***_***3***_)-CO; m, 3H, C***H***_***3***_–CH–CO–NH); 0.96–0.92 (t, 3H, (CH_2_)_2_–CH_2_–C***H***_***3***_). ^1^H NMR spectrum is shown in Figure S1. ^1^H NMR: (con.% = ≥99; DP = 50). SEC (THF): (*M̅*_n_ = 10 600 g mol^–1^; *Đ* = 1.10).

### Synthesis of *N*-*t*-Butoxycarbonyl-*N*′-acryloyl-1,2-diaminoethane (BocAEAM)

The synthesis of the BocAEAm monomer was performed following a procedure previously reported in the literature and described in our previous reports.^48,50^

### Synthesis of PLLA_50_-*b*-P(BocAEAM)*_m_*-*co*-P(NIPAM)*_I_* via PI-RAFT Polymerization

In a typical experiment, PLLA_50_-PABTC–OH, BocAEAM (128 mg, 0.6 mmol), and NIPAM (158 mg, 1.4 mmol) were dissolved in 1,4-dioxane (1713 μL) using a glass tube equipped with a septum. The resulting solution was purged through an argon stream for 5 min. Then, the mixture solution was placed into a photoreactor to induce radical initiation via blue light at λ = 455 nm with an approximate intensity of 204 mW cm^–2^ for 5 h. Afterward, the block copolymer was precipitated three times in water and collected by centrifugation as a white solid, followed by drying under a vacuum.

### PLLA_50_-*b*-P(BocAEAM)_133_-*co*-P(NIPAM)_181_

PLLA_50_-PABTC–OH (50.0 mg, 0.0067 mmol). The ^1^H NMR spectrum is shown in Figure S2. ^1^H NMR (DMSO-*d*_6_): (con.% = 98; DP = 314). SEC (THF): (*M̅*_n_ = 34,500 g mol^–1^; *Đ* = 1.06).

### PLLA_50_-*b*-P(BocAEAM)_268_-*co*-P(NIPAM)_342_

PLLA_50_-PABTC–OH (25.0 mg, 0.0033 mmol). The ^1^H NMR spectrum is shown in Figure S3. ^1^H NMR (DMSO-*d*_6_): (con.% = 98; DP = 610). SEC (THF): (*M̅*_n_ = 44 300 g mol^–1^; *Đ* = 1.14).

### Preparation of Polydisperse Nano-objects by the CDSA of PLLA_50_-*b*-P(BocAEAM)_133_-*co*-P(NIPAM)_181_

30 mg of PLLA_50_-*b*-P(BocAEAM)_133_-*co*-P(NIPAM)_181_ was solubilized in 600 μL of DMSO (10%) using a screw-cap glass vial. Then, 5400 μL of EtOH (90%) was gradually added to achieve a polymer concentration of 5 mg mL^–1^. The reaction mixture was heated at 70 °C using an oil bath for 3 h. Subsequently, the temperature was decreased slowly to room temperature over an extended period of 3 h. After 5 days of aging the solution, the formed micelles were characterized by AFM, as shown in Figure S4.^52^

### Boc-Deprotection Process of Polydisperse Nanostructures

A 450 μL portion of the prepared nano-objects in DMSO/EtOH (v/v = 1:9) was gently transferred into a screw-cap glass vial using a micropipette. Then, the dispersion was incubated with 23 μL of TFA (5%) without stirring for 1 h at room temperature. Afterward, 200 μL of the treated nanostructures was withdrawn for ^1^H NMR characterization to determine the percentage of the deprotection, and 200 μL was taken for DLS analysis to measure the average size. The experiment was repeated by incubating the dispersion with varied concentrations of TFA (10, 15, 20, and 25% equal to 45, 68, 90, and 113 μL, respectively) for 24 h and 1 week. The relevant ^1^H NMR data and DLS analysis of the Boc deprotection process are summarized in Table S1.

### Boc-Deprotection Process of the Diblock Copolymer of PLLA_50_-*b*-P(BocAEAM)*_m_*-*co*-P(NIPAM)*_I_*

250 mg of PLLA_50_-*b*-P(BocAEAM)*_m_*-*co*-P(NIPAM)*_I_* was dissolved in 2.5 mL of TFA using a screw-cap glass vial. The mixture solution was stirred at room temperature for 20 min. Afterward, the block copolymer was precipitated in Et_2_O and collected by centrifugation. The liquid fraction was discarded by pouring it out of the centrifuge tube. To remove the acidic residuals, the polymer was resuspended by adding fresh Et_2_O and centrifuged again, and the step was repeated two times. Then, the polymer was isolated as a white solid and dried overnight under vacuum. This experiment was performed similarly for both synthesized BCPs with a DP of 314 and 610.^55^

### Preparation of Polydisperse Nanoplatelets by the CDSA of the Deprotected PLLA_50_-*b*-P(BocAEAM)_133_-*co*-P(NIPAM)_181_

130 mg of the deprotected polymer was dissolved in 5.2 mL of DMSO (20%) in a Schlenk tube. Then, 20.8 mL of MeOH (80%) was added dropwise by using a funnel to obtain a polymer concentration of 5 mg mL^–1^. The reaction mixture was heated at 60 °C using an oil bath for 3 h. Then, the temperature was dropped slowly to room temperature over 2.5 h. After 1 week of aging the solution, the obtained platelets were purified by dialysis against PBS (9.55 mg mL^–1^, pH of 7.5) using a dialysis membrane bag with a molecular weight cutoff (MWCO) of 3.5 kDa. Dialysis was performed at room temperature in a beaker for 2 days with replacing the PBS three times to ensure removing the solvent residuals. The formation of platelets was proved by AFM (Figures S7 and S8), and the average size of the assembled particles was measured by DLS (Figure S10).

### Preparation of Polydisperse Nanospheres by the CDSA of the Deprotected PLLA_50_-*b*-P(BocAEAM)_133_-*co*-P(NIPAM)_181_

20 mg of the deprotected polymer was solubilized in 400 μL of DMSO (10%) using a screw-cap glass vial. Then, 3.6 mL of ultrapure water (90%) was slowly introduced by using a syringe to obtain a polymer concentration of 5 mg mL^–1^. The resultant mixture was heated at 85 °C using an oil bath for 4 h. Then, the temperature was dropped gradually to room temperature over 3.5 h. After 3 days of aging, the obtained spheres were purified by dialysis against PBS (9.55 mg mL^–1^, pH of 7.5) using a dialysis membrane bag with a molecular weight cutoff (MWCO) of 3.5 kDa. Dialysis was performed at room temperature in a beaker for 1 day with replacing the PBS two times. The formation of spherical particles was proved by AFM (Figures S7 and S9), and the average size of the assembled particles was measured by DLS (Figure S10).

### Self-Assembly Process of the Deprotected PLLA_50_-*b*-P(BocAEAM)_268_-*co*-P(NIPAM)_342_

5 mg of the deprotected polymer was solubilized in 200 μL of DMSO (20%) using a screw-cap glass vial. Then, 800 μL of MeOH (80%) was slowly introduced by using a syringe. The mixture was heated at 60 °C using an oil bath for 3 h. Then, the temperature was dropped gradually to room temperature over 2.5 h. After 5 days of aging, the formed micelles were characterized by AFM (Figure S11) and DLS (Figure S12).

### GIWAXS Measurements

The GIWAXS experiments were performed using a SAXSLAB laboratory setup (Retro-F) (Copenhagen, Denmark) equipped with an AXO microfocus X-ray source (Dresden, Germany) and an AXO multilayer X-ray optics (ASTIX) as a monochromator for Cu Kα radiation (λ = 0.15418 nm). A DECTRIS PILATUS3 R 300 K detector (Daettwil, Switzerland) was used to record the 2D GIWAXS patterns. The measurements were performed in reflection geometry in a vacuum at room temperature, and the distance from the sample to the detector was approximately 92 mm. The GIWAXS detector images were converted into the reciprocal space maps with two components, *q*_z_ and *q*_p_, being perpendicular and parallel to the sample surface, respectively. Due to the special geometry of the measurements, a certain area of the reciprocal space along the *q*_*z*_ axis was not accessible and appeared as a blank arc. Two additional blank vertical strips arose at the positions where two of the three adjacent parts of the detector meet and were inactive regions of the detector.

### Minimum Inhibitory Concentration (MIC_50_)

To determine the minimum inhibitory concentration of the samples, two Gram-negative bacteria, *E. coli* (*E. coli*, ATCC 25922), *P. aeruginosa* (*P. aeruginosa*, ATCC 10145), and one Gram-positive bacteria, methicillin-resistant *S. aureus* (MRSA; ATCC 43300), were investigated. Cell culture was prepared by inoculation of a single colony of the respective bacterial strains in Mueller–Hinton broth medium (MHB, 5 mL). The solution was incubated overnight at 37 °C. The concentration of cells was evaluated via measuring optical density at 600 nm (OD_600_). Then, cells were diluted with MHB to achieve an OD_600_ of 0.1. To the bacterial suspension, medium was added in a ratio of 1:5000 to prepare the final bacterial suspension. Next to this, APs were diluted with MHB and a serial dilution in a 96-well plate was performed (three determinations of each concentration; range of concentration: 2048–1 μg mL^–1^, each well containing 100 μL of the polymer solution). Afterward, the bacterial suspension (100 μL) was added to the well plate. As a negative control, wells containing only medium were used, and wells containing 100 μL of bacterial suspension and 100 μL of MHB served as a positive control. By measuring OD_600_, the growth of bacteria was determined and normalized using positive and negative controls. MIC_50_ was calculated using a Hill1 Fit of OriginPro 2021 for *E. coli* and was set as the lowest concentration with less than 50% absorption (at 600 nm) for *P. aeruginosa*.

### Blood Compatibility Tests

To test hemocompatibility, defibrinated sheep blood was used, and red blood cells (RBCs) were isolated by centrifugation at 4500 rpm for 1 min. Afterward, RBCs were washed two times via centrifugation with phosphate-buffered saline (PBS), which served as medium and were diluted in a ratio of 1:15 with medium. This blood suspension was used for the measurement. The samples were dissolved in PBS and a serial dilution was performed using a well plate (each well containing 100 μL of the respective polymer solution; three determinations of each concentration). The range of concentration was 4096–8 μg mL^–1^ for diamond-shaped nanoparticles and 2048–8 μg mL^–1^ for nanospheres. Then, 100 μL of the blood suspension was added to the well plate. Wells containing RBCs and Triton X solution (1% in PBS) were used as a positive control, and wells containing only PBS and RBCs served as a negative control. After this, the well plate was incubated for 1 h at 37 °C. To separate RBCs from the suspension, the well plate was centrifuged for 5 min at 500 G. Accordingly, 100 μL of the supernatant of each well was added to another well plate and measured via UV absorption at 544 nm. Results were normalized using negative and positive controls. Hemolysis (Hc_10_) was defined as the highest concentration, which induced less than 10% lysis of RBCs.

### Preparation of Liposomes

*E. coli*-mimicking liposomes used in this study for dye leakage measurements were prepared by thin film hydration and extrusion methods following a protocol previously reported.^47,56^ Briefly, 2-oleoyl-1-palmitoyl-*sn*-glycero-3-phosphoethanolamine (POPE) (8 mg, 11.14 μmol) and 2-oleoyl-1-palmitoyl-*sn*-glycero-3-phospho-rac-(1-glycerol) sodium salt (POPG) (2 mg, 2.59 μmol) were mixed in CHCl_3_ (1 mL) in a 25 mL round-bottom flask. The lipid mixture was treated under a vacuum to form a thin-film lipid. Then, a subsequent hydration with 1 mL of calcein solution (0.4 mM) was performed with continuous stirring for 1 h at room temperature. To maximize the dye encapsulation, five freeze–thaw cycles in liquid nitrogen for 5 min and then in a water bath at 25 °C for 10 min were applied. The vesicles were extruded 15 times successively by using polycarbonate membranes of 400 nm pore size. The extruded liposomes were purified two times to remove nonencapsulated calcein by centrifugation at 4000 rpm overnight using an Amicon Ultra-15, PLTK Ultracel-PL membrane (30KDa MWCO) with PBS as dispersing media. The concentrated liposomal suspension was recovered and stored at 4 °C for use within 1 week.

### Dye Leakage Experiment

A time-based fluorescence experiment was performed for each polymer concentration: 2 mL of calcein-loaded liposomes (diluted 80 times with PBS) was placed in a quartz cuvette with continuous stirring. A baseline of calcein fluorescence without polymer addition was normalized for each sample. 20 μL of polymer solutions (in PBS) of various concentrations was added to the cuvette 100 s after the start of the run and left to incubate with the liposomes. After 2200 s when equilibrium was reached, 20 μL of Triton X (20%) in PBS was added as a positive control to completely disrupt the liposomes and therefore to determine the fluorescence intensity corresponding to 100% dye leakage. The measured fluorescence intensity was normalized into percentage leakage activity, *Y*, using eq 1
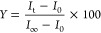
1where *I*_o_ is the fluorescence intensity *I*_t_ before the addition of the polymer samples and *I*_∞_ is the *I*_t_ after the addition of Triton-X (or the maximum dye leakage at higher polymer concentration). To determine the 50% effective concentration of the polymer inducing dye leakage, EC_50_, the maximum dye leakage percentages reached before the addition of Triton-X was plotted versus the respective polymer concentrations.

## Supporting Information Available

NMR spectra; AFM micrographs; DLS size distributions; GIWAXS data; polarization microscopy; growth inhibition curves; and hemolysis data (PDF)
