# Supplementary material for: Antibacterial Nanoplatelets via Crystallization-Driven Self-Assembly of Poly(l-lactide)-Based Block Copolymers
Source: Biomacromolecules. 2024 Aug 6;25(9):6103–14. doi: 10.1021/acs.biomac.4c00767 (PMC11388454; doi:10.1021/acs.biomac.4c00767)
Supplement: Supplementary file 1 — bm4c00767_si_001.pdf [file bm4c00767_si_001.pdf]

Supporting Information

Antibacterial Nanoplatelets via Crystallization-Driven Self-Assembly of Poly(L-lactide)-based Block Copolymers

*Ahmad Alsawaf, Anne-Catherine Lehnen, Oleksandr Dolynchuk, Alain M. Bapolisi, Christina Beresowski, Alexander Böker, Ilko Bald, Matthias Hartlieb**

A. Alsawaf, A. Lehnen, A. Bapolisi, C. Beresowski, A. Böker, I. Bald, M. Hartlieb
Institute of Chemistry, University of Potsdam, Karl-Liebknecht-Str. 24-25, 14476 Potsdam, Germany

Oleksandr Dolynchuk
Experimental Polymer Physics, Martin Luther University Halle-Wittenberg, Von-Danckelmann

Platz 3, 06120 Halle, Germany

A.-C. Lehnen, A. Böker, M. Hartlieb
Fraunhofer Institute for Applied Polymer Research (IAP), Geiselbergstraße 69, 14476 Potsdam, Germany

*E-mail: mhartlieb@uni-potsdam.de

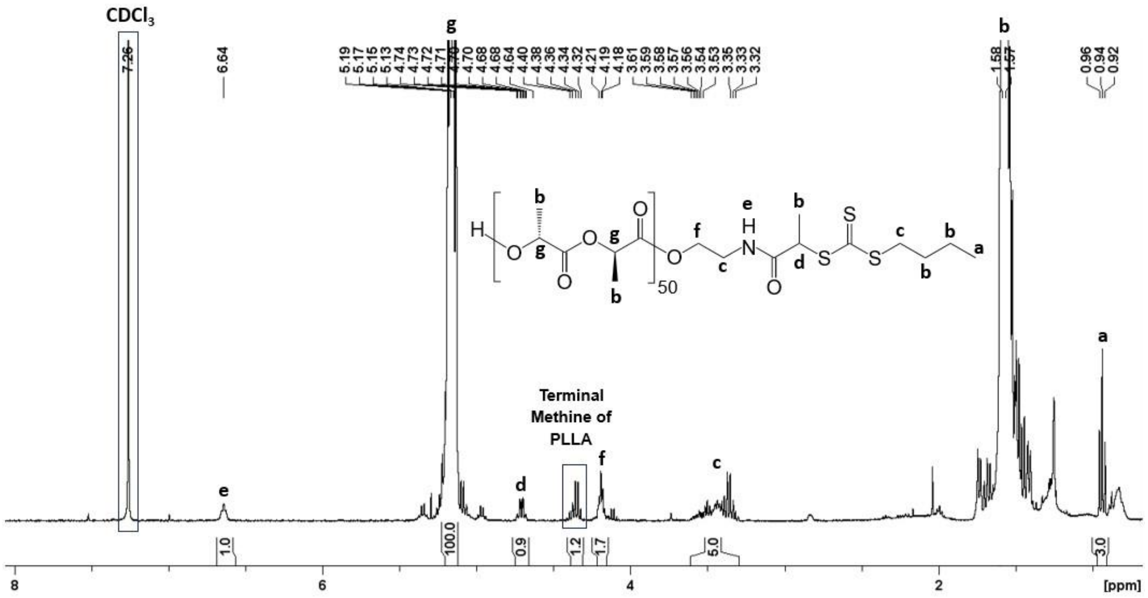

Figure S1. ^{1}H NMR (400 MHz) spectrum of a macro-CTA of $PLLA_{50}$-PABTC-OH in $CDCl_3$.

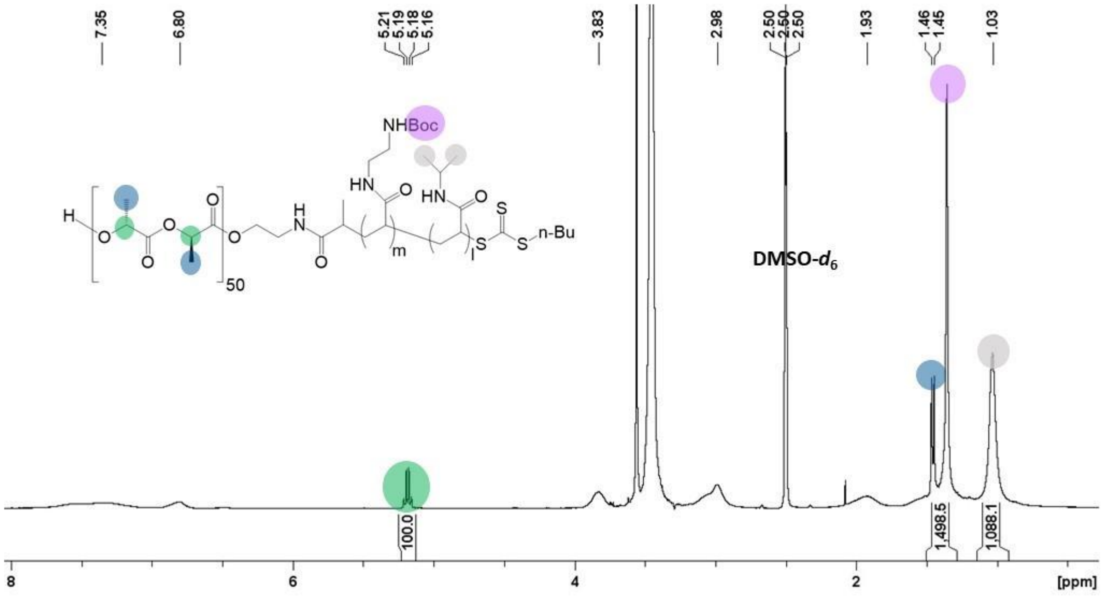

Figure S2. ^{1}H NMR (400 MHz) spectrum of diblock copolymers of $PLLA_{50}$-*b*-$PBocAEAm_{133}$-*co*-$PNiPAAm_{181}$ targeted via PI-RAFT polymerization using a macro-initiator of $PLLA_{50}$-PABTC-OH in DMSO-d_6.

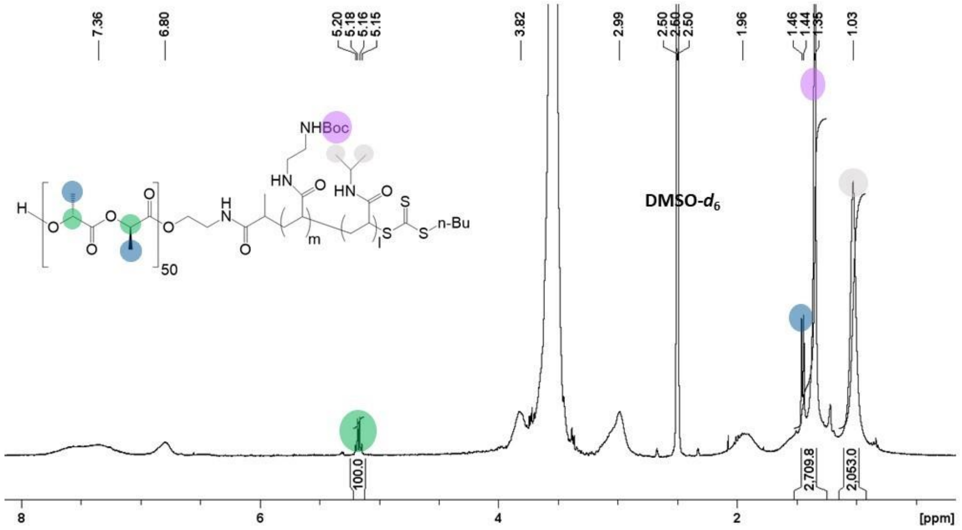

Figure S3. ^{1}H NMR (400 MHz) spectrum of diblock copolymers of PLLA$_{50}$-*b*-PBocAEAm$_{268}$-*co*-PNiPAAm$_{342}$ targeted via PI-RAFT polymerization using a macro-initiator of PLLA$_{50}$-PABTC-OH in DMSO-d_6.

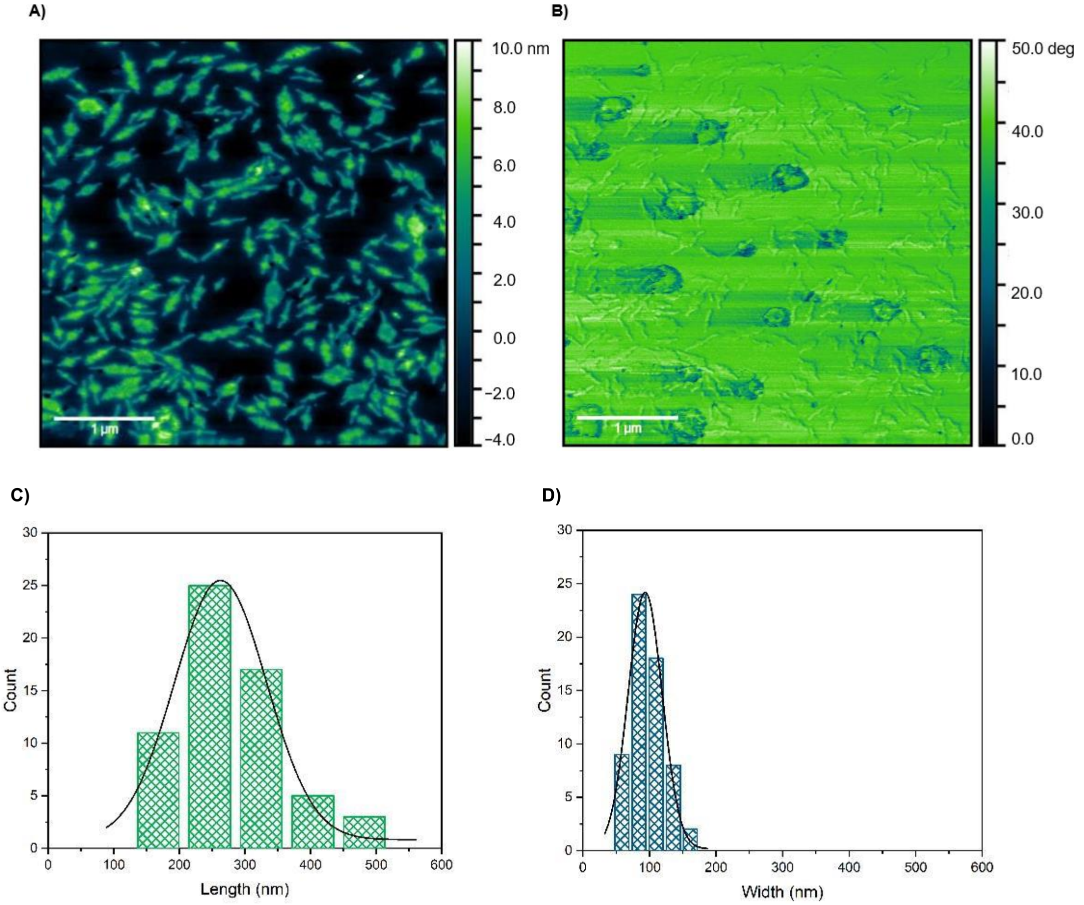

Figure S4. (A) AFM height image of diblock copolymers of PLLA$_{50}$-*b*-PBocAEAm$_{133}$-*co*-PNiPAAm$_{181}$ of polydisperse fiber-like micelle at a concentration of 0.5 mg mL^{-1}. (B) AFM phase image of (A). (C) Contour length histogram from (A). (D) Contour width histogram from (A).

Table S1. Summary of ^{1}H NMR data and DLS analysis of Boc deprotection process from the polydisperse fiber-like micelle of $PLLA_{50}$-*b*-$PBocAEAm_{133}$-*co*-$PNiPAAm_{181}$ through varying the concentration of TFA at RT or 40 °C within different reaction times.

Ex. No.	time (h or day)	T (°C)	TFA (%)	Boc [a] (%)	Deprotection [a] (%)	DP_{PLLA} [a] (-)	Z-Average [b] (nm)	PDI [b] (-)
**A**	1 h	RT	5	98	2	50	180	0.05
**B**	24 h	RT	5	96	4	48	178	0.05
**C**	24 h	RT	10	94	6	49	180	0.02
**D**	24 h	RT	15	92	8	42	174	0.04
**E**	24 h	40	10	86	14	46	170	0.06
**F**	7 days	RT	5	96	4	46	173	0.01
**G**	7 days	RT	10	87	13	44	175	0.02
**H**	7 days	RT	15	86	14	47	171	0.03
**I**	7 days	RT	20	85	15	45	171	0.05
**J**	7 days	RT	25	78	22	43	166	0.0002
**K**	1 h	RT	50	86	14	41	-	-
**L**	1 h	RT	75	80	20	39	-	-

[a] Determined via ^{1}H-NMR spectroscopy in DMSO-d_6. [b] Measured via dynamic light scattering (DLS) in EtOH.

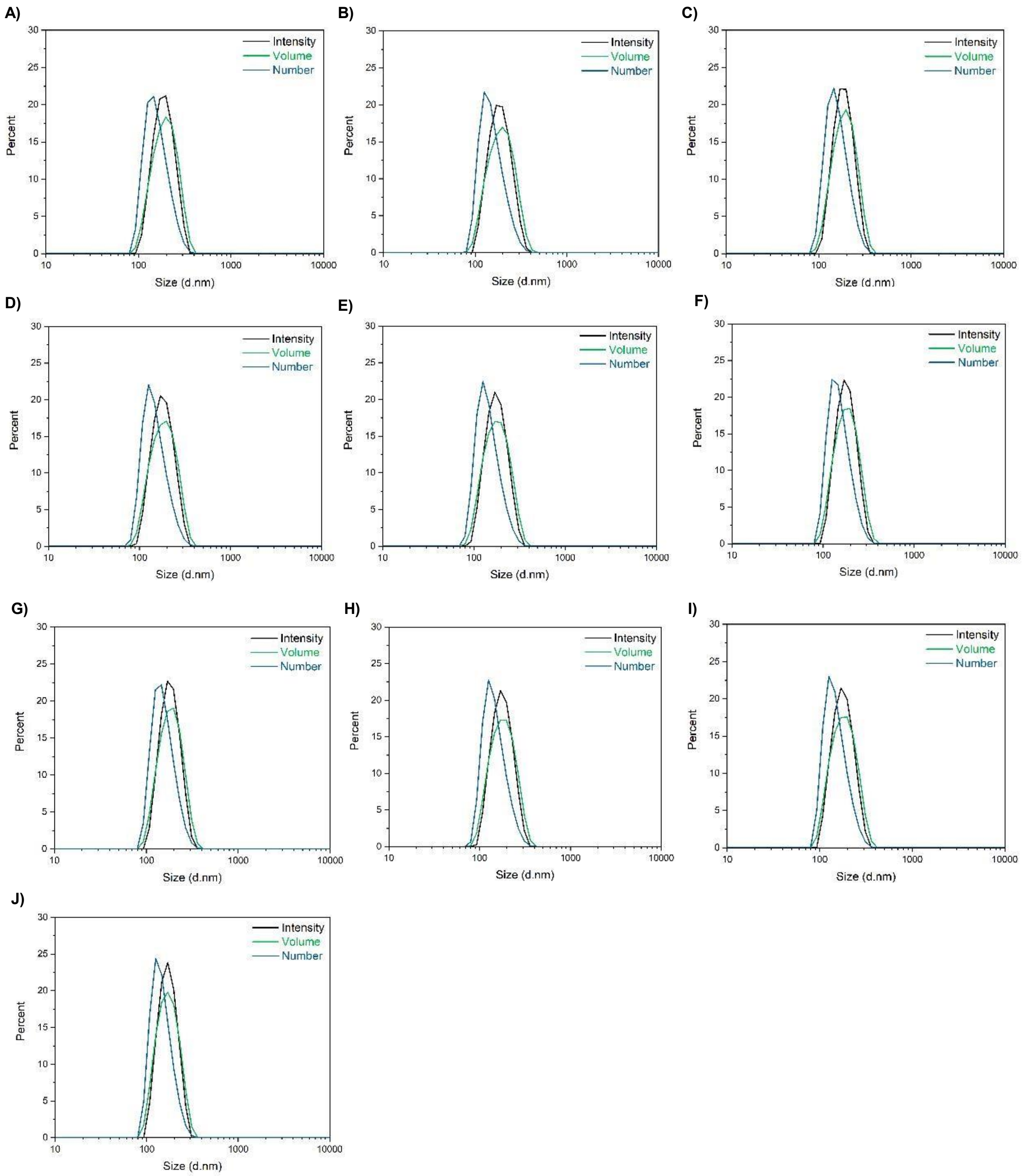

Figure S5. DLS analysis of Boc deprotection process from the polydisperse fiber-like micelle of $PLLA_{50}$-*b*-$PBocAEAm_{133}$-*co*-$PNiPAAm_{181}$ at a concentration of 1 mg mL^{-1}. (A) After treatment with 5%TFA for 1 h at RT. (B) After treatment with 5%TFA for 24 h at RT. (C) After treatment with 10%TFA for 24 h at RT. (D) After treatment with 15%TFA for 24 h at RT. (E) After treatment with 10%TFA for 24 h at 40 °C. (F) After treatment with 5%TFA for 7 days at RT. (G) After treatment with 10%TFA for 7 days at RT. (H) After treatment with 15%TFA for 7 days at RT. (I) After treatment with 20%TFA for 7 days at RT. (J) After treatment with 25%TFA for 7 days at RT.

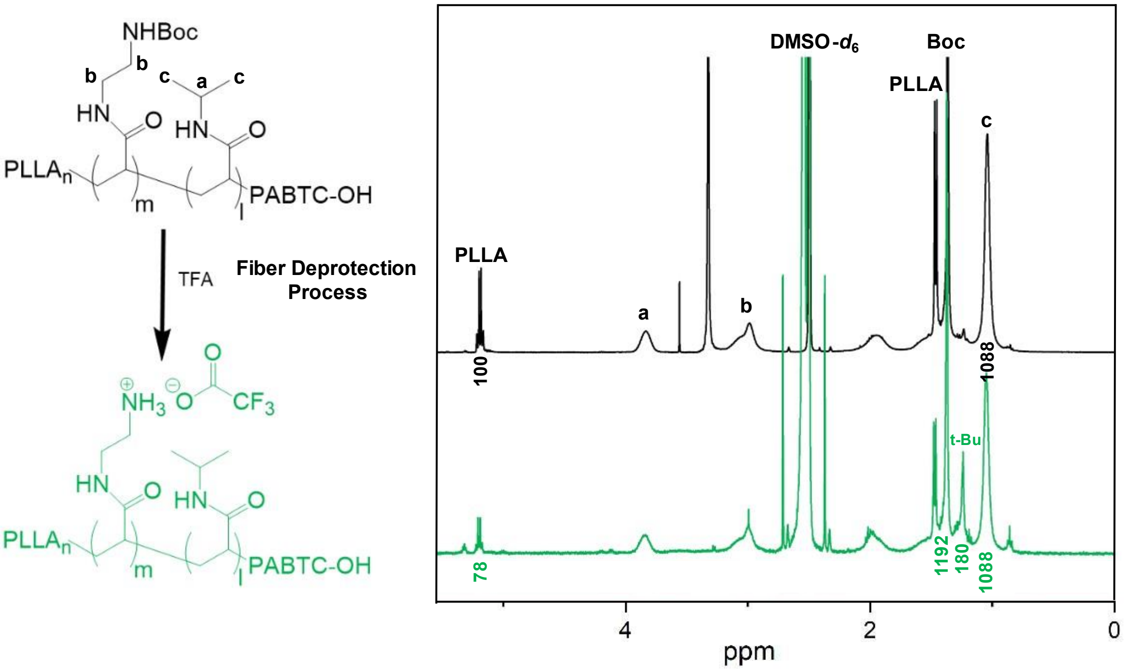

Figure S6. Fiber deprotection process of $PLLA_{50}$-*b*-$PBocAEAm_{133}$-*co*-$PNiPAAm_{181}$ through treatment with 75% TFA at room temperature for 1 h proved by ^{1}H NMR analysis in DMSO-d_6.

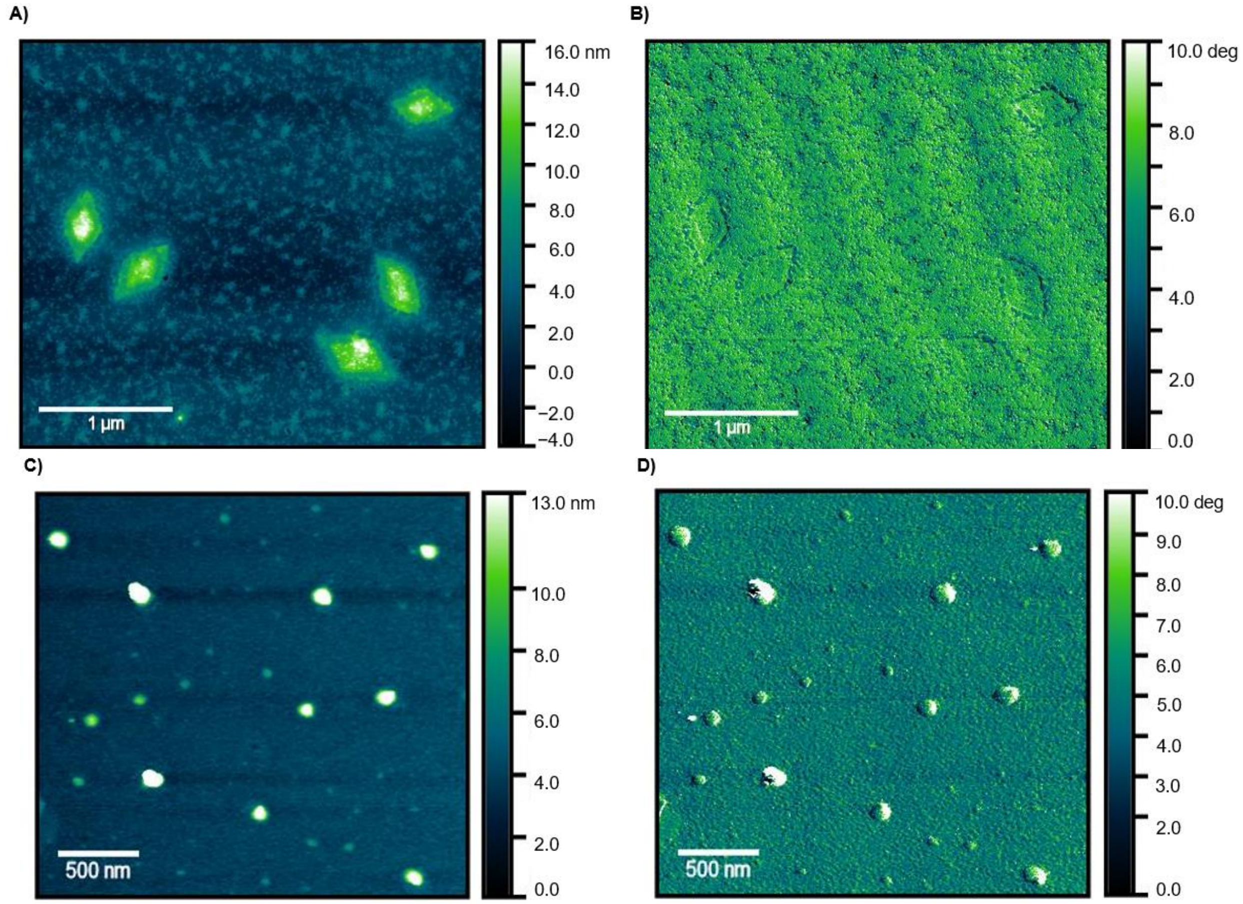

Figure S7. (A) AFM height image of the deprotected BCPs of $PLLA_{50}$-*b*-$PBocAEAm_{133}$-*co*-$PNiPAAm_{181}$ of polydisperse cationic diamond nanoplatelets obtained after dialysis against PBS at pH of 7.5 at a concentration of 0.5 mg mL^{-1}. (B) AFM phase image of (A). (C) AFM height image of the deprotected BCPs of $PLLA_{50}$-*b*-$PBocAEAm_{133}$-*co*-$PNiPAAm_{181}$ of polydisperse cationic nanospheres obtained after dialysis against PBS at pH of 7.5 at a concentration of 0.5 mg mL^{-1}. (D) AFM phase image of (C).

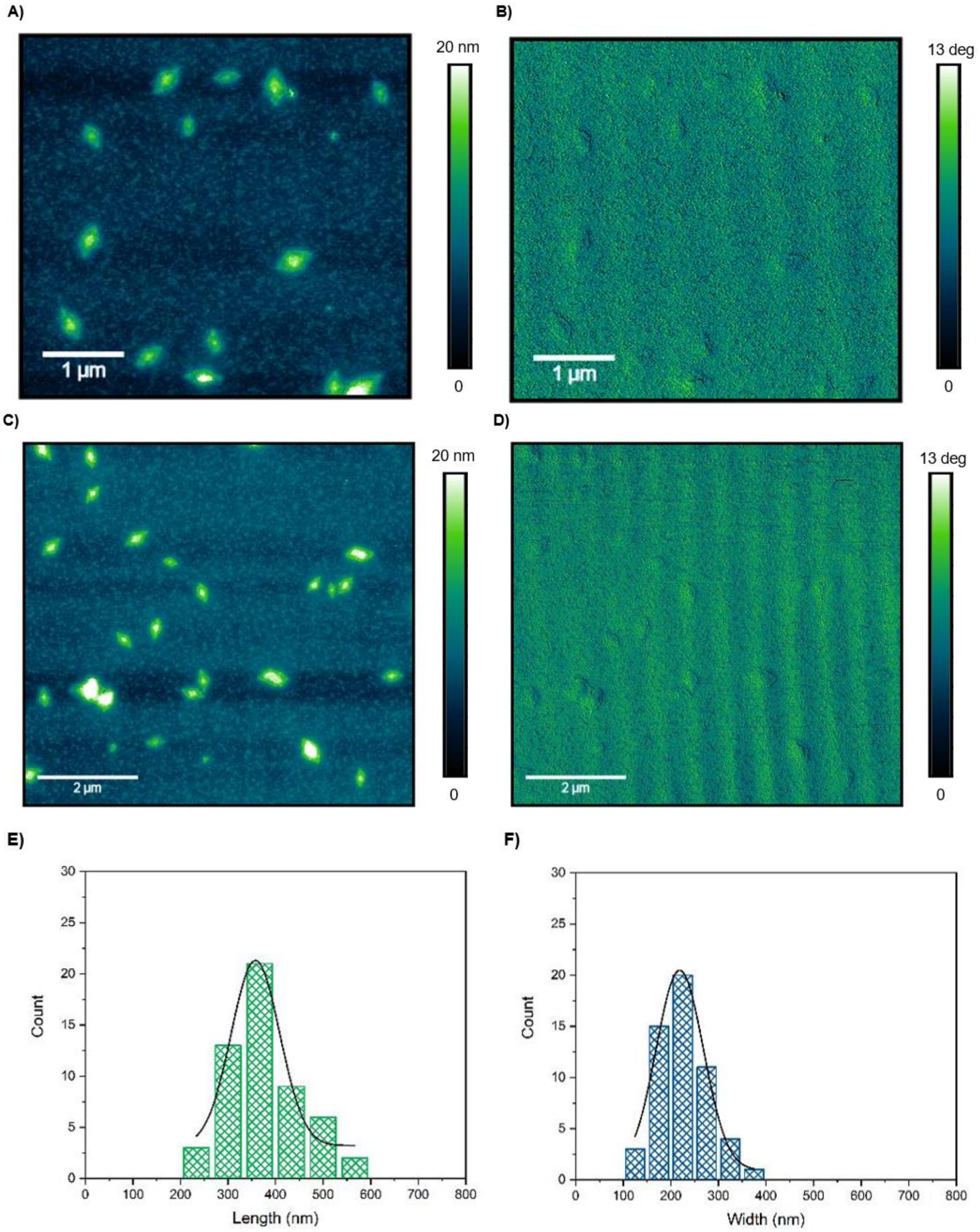

Figure S8. (A) and (C) AFM height image of the deprotected BCPs of $PLLA_{50}$-*b*-$PBocAEAm_{133}$-*co*-$PNiPAAm_{181}$ of polydisperse cationic diamond nanoplatelets measured after dialysis against PBS at pH of 7.5 at a concentration of 0.5 mg mL^{-1}. (B) AFM phase image of (A). (D) AFM phase image of (C). (E) Contour length histogram from (A) and (C). (F) Contour width histogram from (A) and (C).

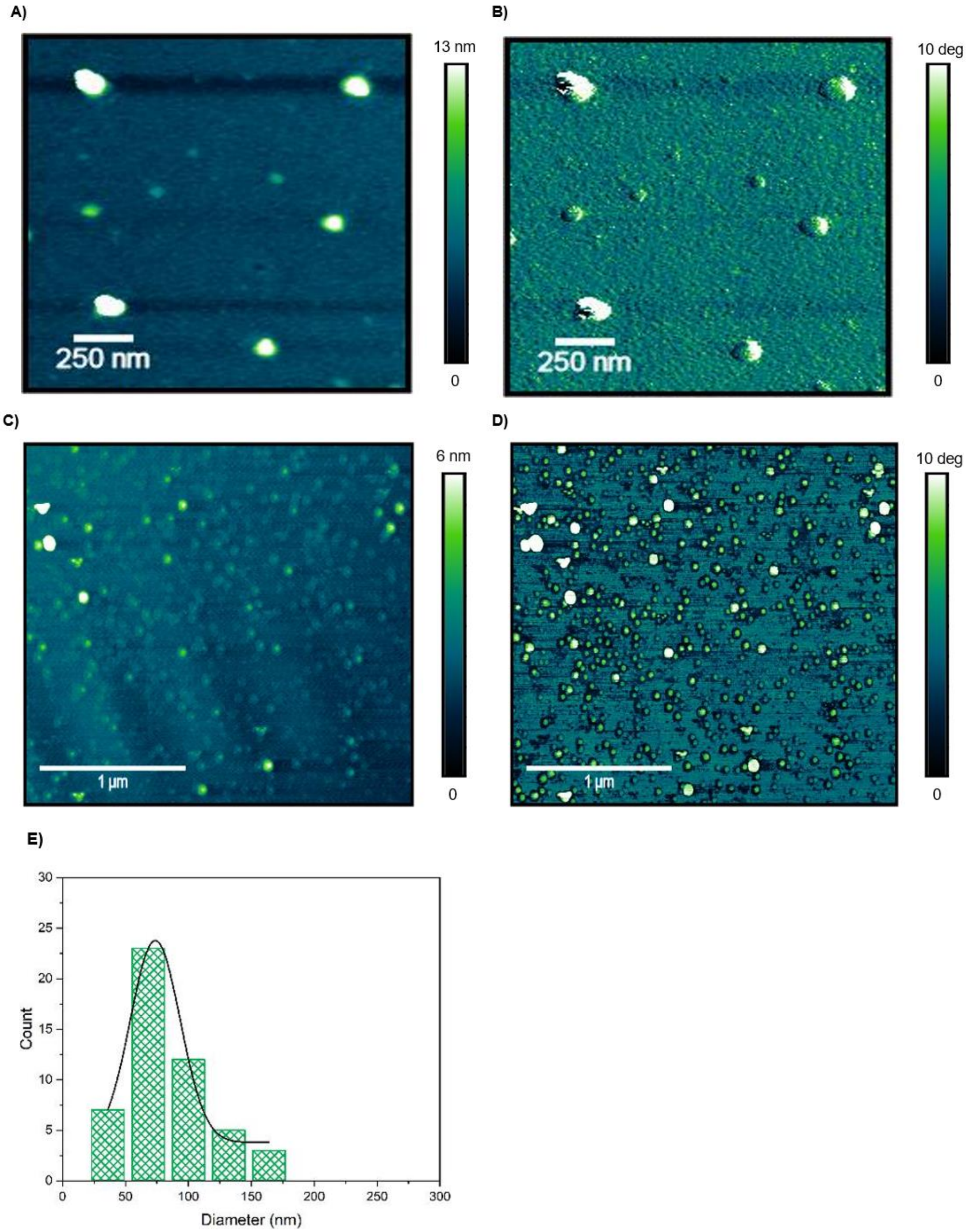

Figure S9. (A) and (C) AFM height image of the deprotected BCPs of PLLA$_{50}$-*b*-PBocAEAm$_{133}$-*co*-PNiPAAm$_{181}$ of polydisperse cationic nanospheres measured after dialysis against PBS at pH of 7.5 at a concentration of 0.5 mg mL^{-1}. (B) AFM phase image of (A). (D) AFM phase image of (C). (E) Contour diameter histogram from (A) and (C).

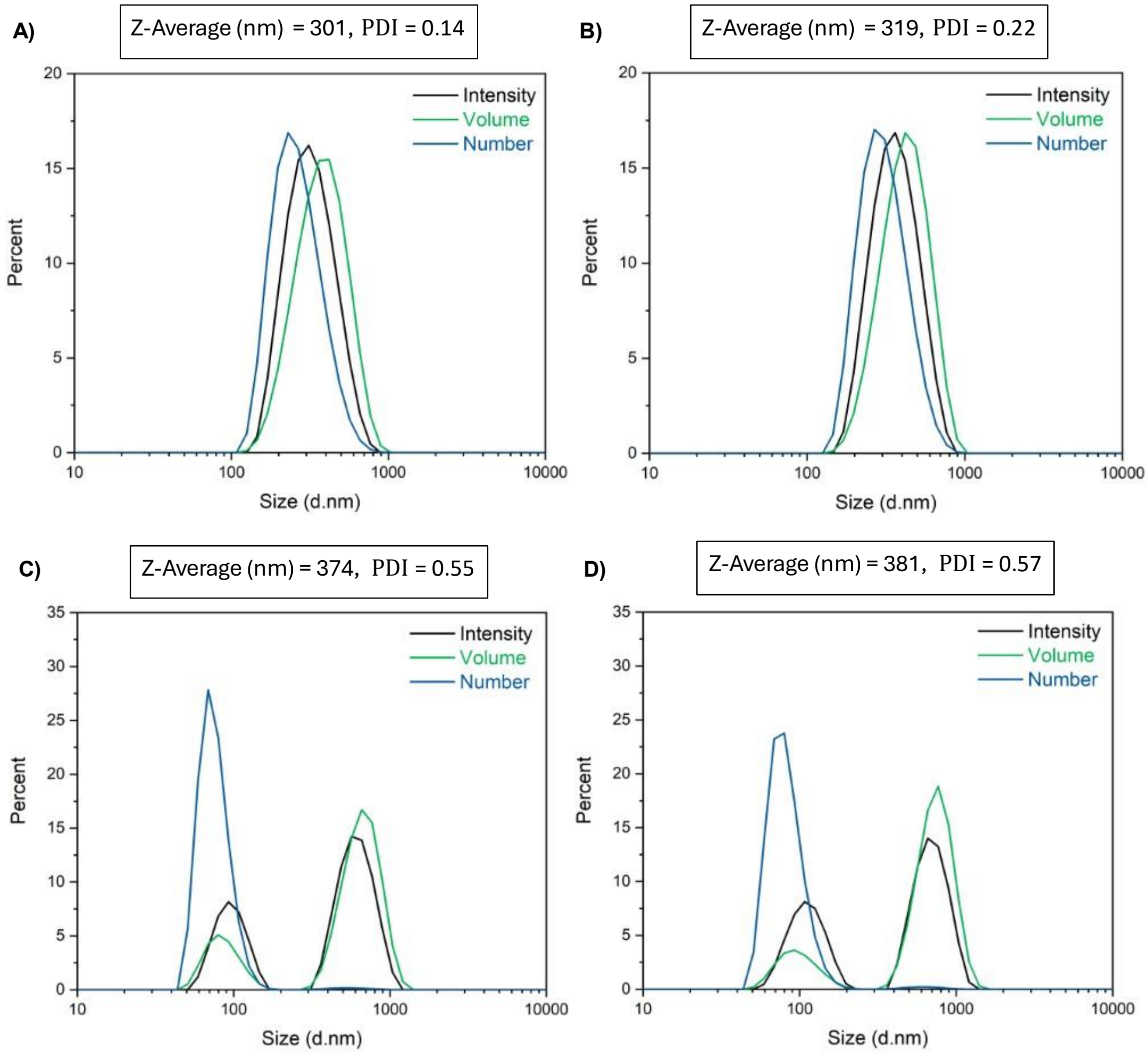

Figure S10. DLS analysis of the self-assembly morphology obtained from the deprotected BCPs of PLLA$_{50}$-*b*-PBocAEAm$_{133}$-*co*-PNiPAAm$_{181}$ at a concentration of 1 mg mL^{-1} after aging at RT. (A) Size distribution by the cationic diamond nanoplatelets obtained after CDSA and measured in MeOH. (B) Size distribution by the cationic diamond nanoplatelets obtained after dialysis against PBS and measured in ultrapure water. (C) Size distribution by the cationic nanospheres obtained after CDSA and measured in ultrapure water. (D) Size distribution by the cationic nanospheres obtained after dialysis against PBS and measured in ultrapure water.

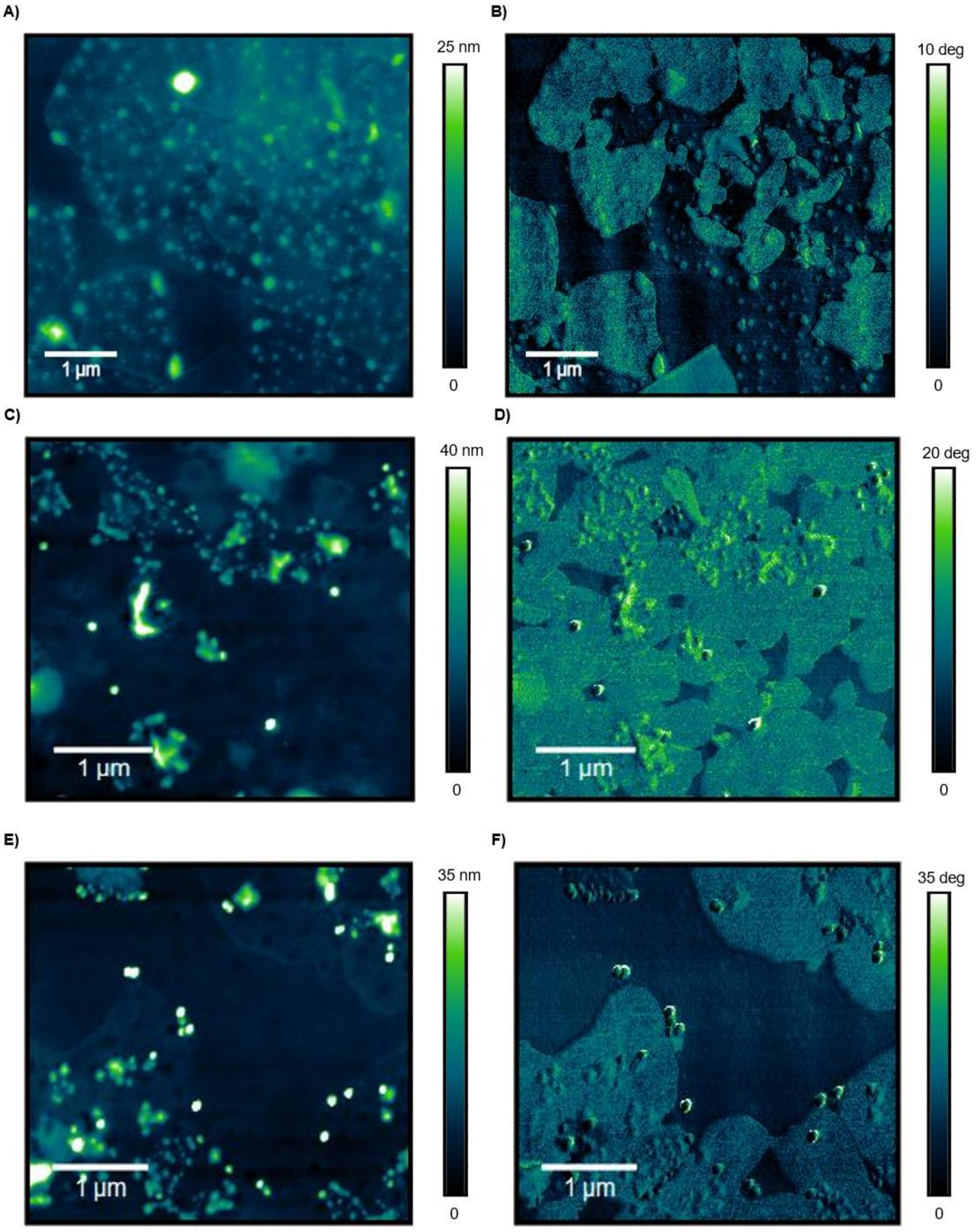

Figure S11. (A), (C), and (E) AFM height image of the self-assembly of the deprotected BCPs of $PLLA_{50}$-*b*-$PBocAEAm_{268}$-*co*-$PNiPAAm_{342}$ in DMSO/MeOH (v:v = 2:8) at 60 °C for 3 h at a concentration of 0.5 mg mL^{-1}. (B), (D), and (F) AFM phase image of (A), (C), and (E) respectively.

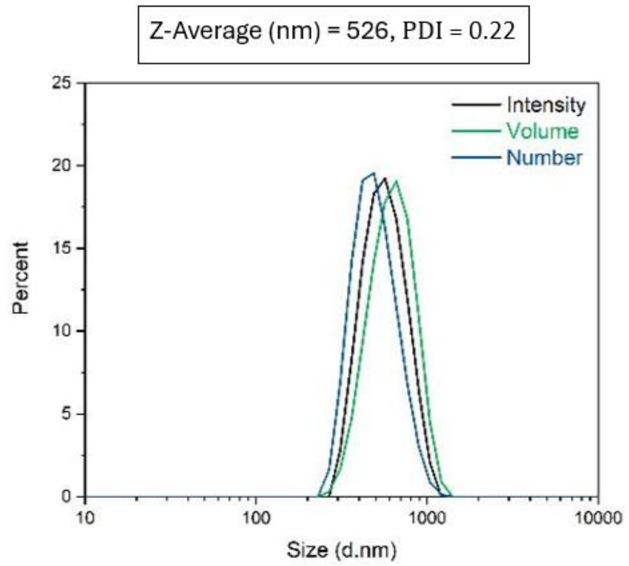

Figure S12. DLS analysis of the self-assembly experiment obtained from of the deprotected BCPs $PLLA_{50}$-*b*-$PBocAEAm_{268}$-*co*-$PNiPAAm_{342}$ at a concentration of 1 mg mL^{-1} after aging at RT.

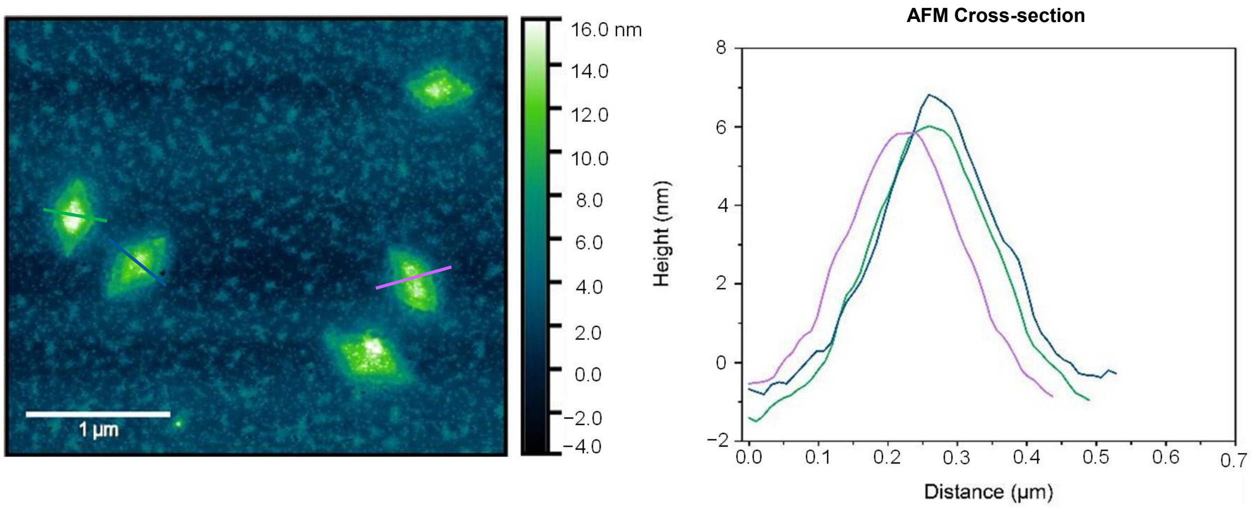

Figure S13. AFM cross sections of nanoplatelets indicating they height of structures.

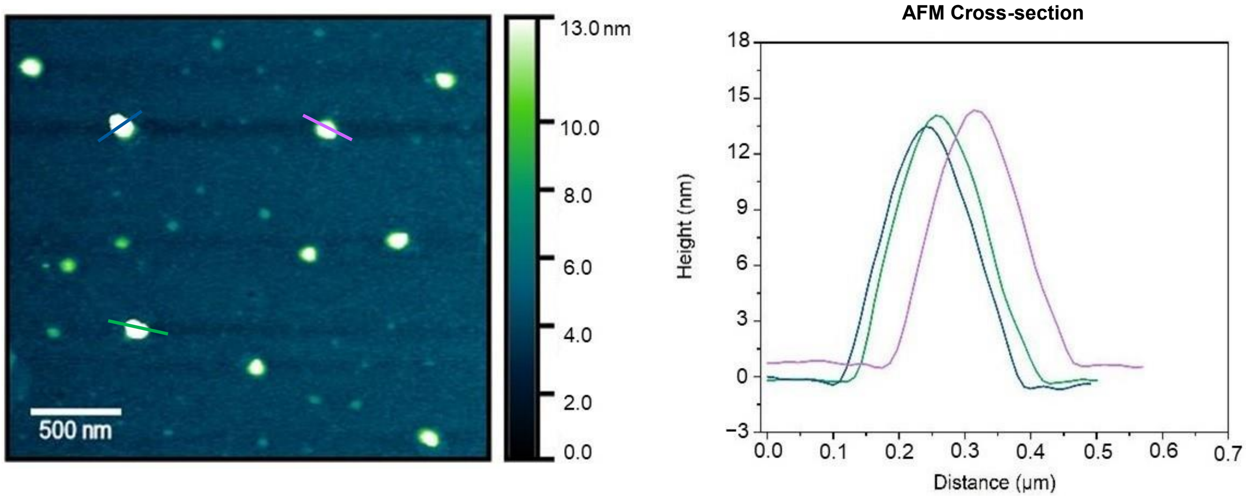

Figure S14. AFM cross sections of nanospheres indicating they height of structures.

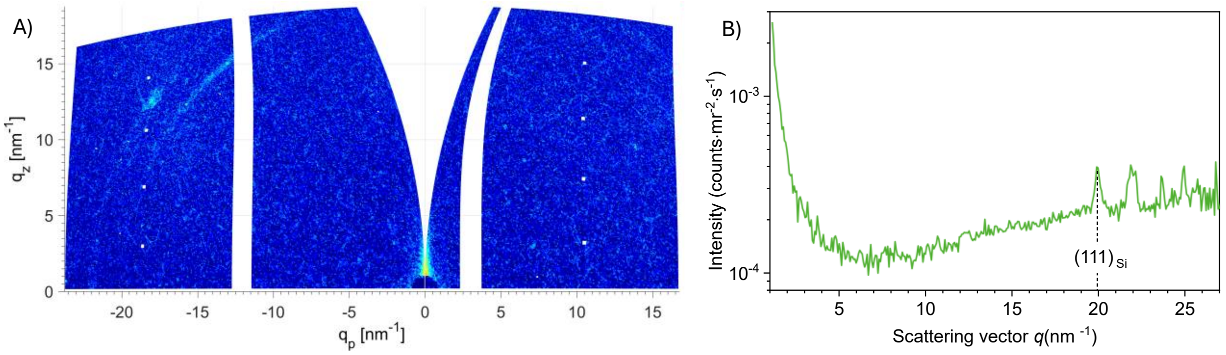

Figure S15. GIWAXS pattern (A) and the respective scattering curve (B) of the nearly empty silicon substrate measured at an incident angle of 0.2°. The scattering curve in (B) was obtained by integrating the intensity in (A) over all azimuthal angles.

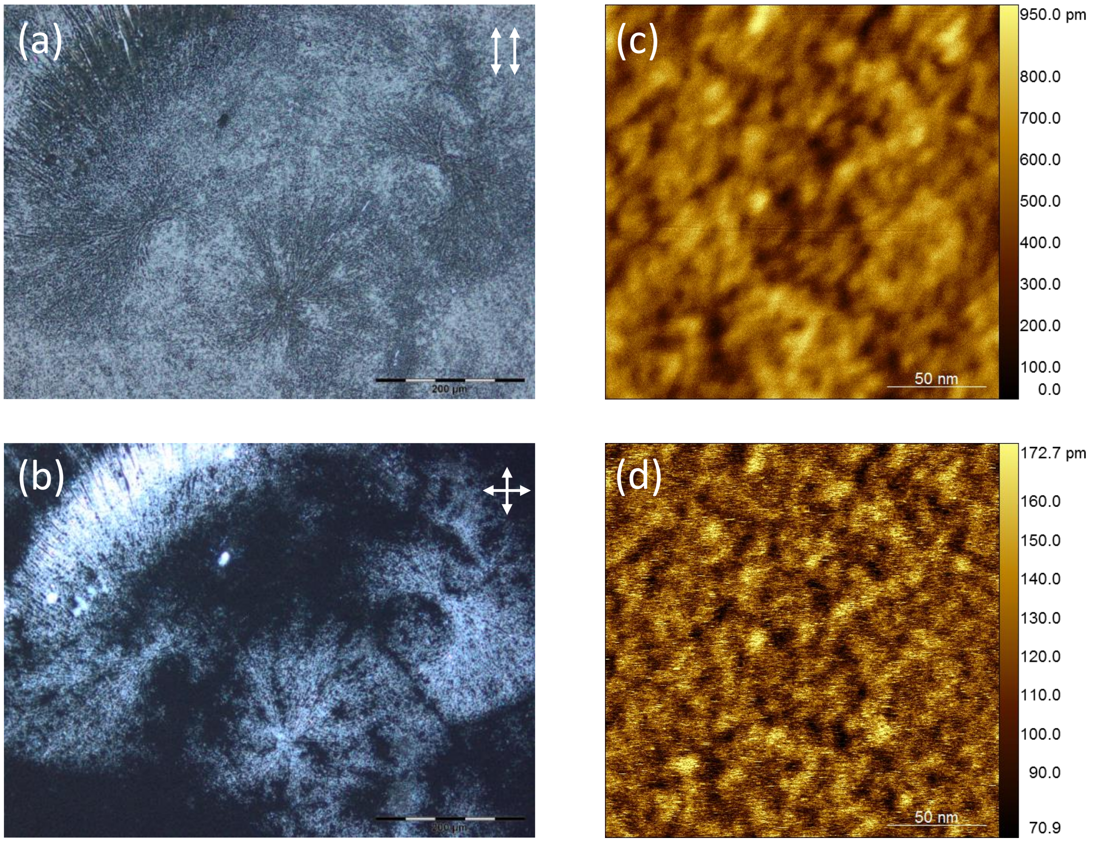

Figure S16. Optical microscopy images (a, b) of the drop casted sample on a silicon substrate under open (a) and crossed (b) polarizers, indicated in the upper right corners of the images. AFM height (c) and deformation (d) images of the drop cast sample on the silicon substrate.

The polarized light optical microscopy image in (b) clearly shows a strong birefringence of the polymer, indicating its semicrystalline nature. Remarkably, one can identify the centers of spherulitic crystal growth in Fig. a - b, suggesting that the polymer crystallization followed the classical route consisting of nucleation and growth steps. The atomic force microscopy images (b - c) show small lamellar crystals of the order of 10 - 20 nm, consistent with the chemical architecture of the polymer chains.

Modell	Hill1	
Gleichung	y = START + (END - START) * x^n / (k^n + x^n)	
Zeichnen	AA097_H	AA097_K with 2.5 mg/mL
START	97.44647 ± 1.13331	99.55792 ± 1.39527
END	21.9362 ± 1.67146	-3.63214 ± 9.41301
k	**32.65151 ± 3.71187**	**153.91001 ± 23.51268**
n	3.38915 ± 0.9547	1.45925 ± 0.32885
Chi-Quadr Reduziert	2.47219	1.71832
R-Quadrat (COD)	0.99481	0.99842
Kor. R-Quadrat	0.99287	0.99783

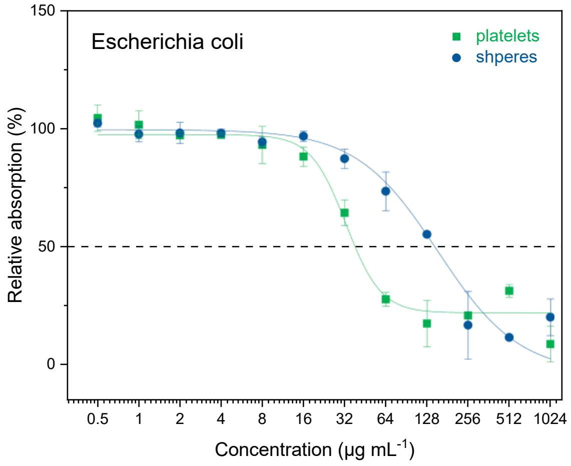

Model	Hill1
Equation	y = START + (END - START) * x^n / (k^n + x^n)
Plot	AA097_H
START	100 ± 0
END	0 ± 0
k	454.21218 ± 178.66879
n	4.10625 ± 1.98769
Reduced Chi-S	3.51226
R-Square (COD	0.9977
Adj. R-Square	0.99748

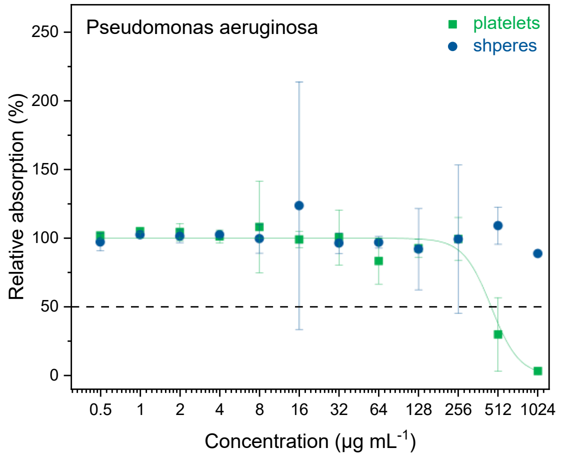

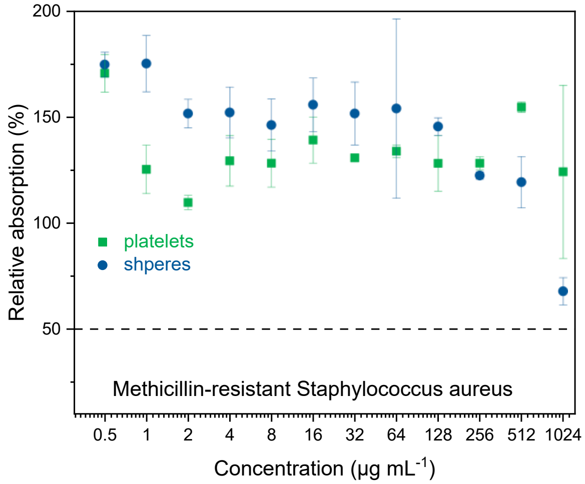

Figure S17. AP concentration-dependent bacterial growth. Absorption was determined via optical density at 600 nm and normalized using positive control (medium with bacteria suspension; 100 %) and negative control (pure medium, 0 %). Mueller-Hinton broth medium (MHB) was used as a culture medium. Values below 50 % were considered as antimicrobial active and a calculation using the Hill1 Fit of OriginPro 2021® was used to determine MIC_{50}. Error bars correspond to triplicates.

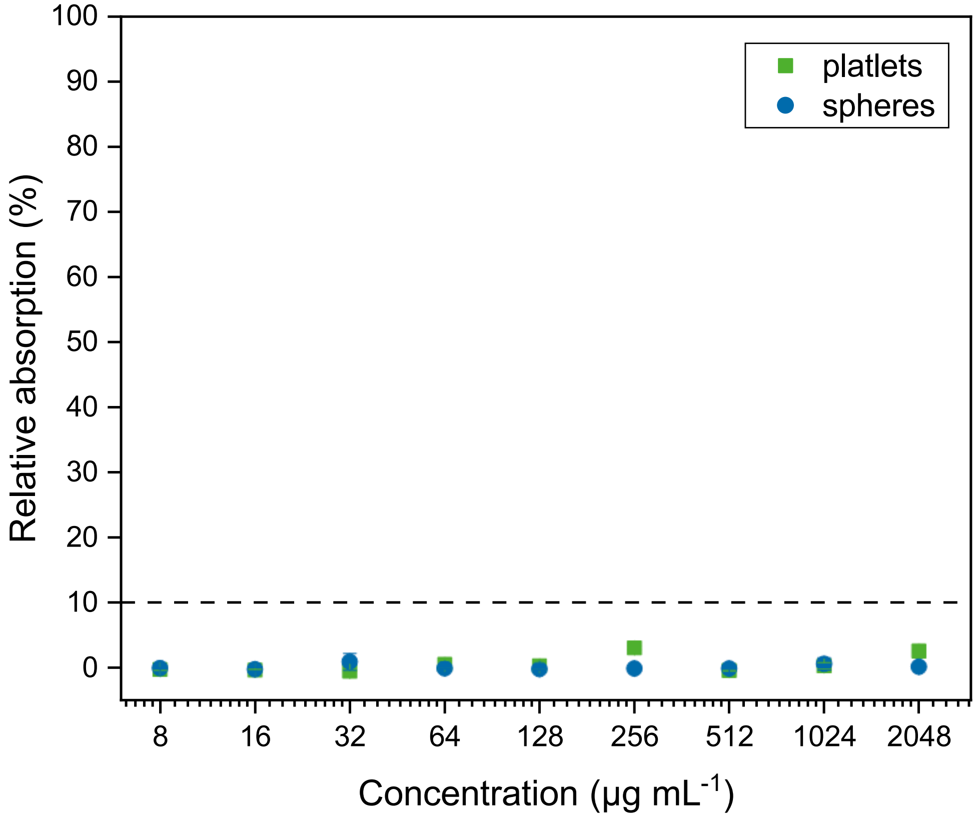

Figure S18. Hemolysis assay of APs. PBS was used as medium and RBCs were threatened with polymer solutions containing different concentrations between 4096 µg mL^{-1} to 8 µg mL^{-1} for diamond-shaped nanoparticles and 2048 µg mL^{-1} to 8 µg mL^{-1} for nanospheres for 1 h at 37 °C. After separation of the cells via centrifugation at 500 G the absorbance of the supernatant was measured using Absorption (544 nm) to analyze hemolysis. RBC solution containing Triton X solution (1 % in PBS) was used as positive control and pure PBS solution served as negative control. A triplicate determination was executed and results below 10 % were set as non-hemolytic.
